# Leptin Gene and Leptin Receptor Gene Polymorphisms in Alcohol Use Disorder: Findings Related to Psychopathology

**DOI:** 10.3389/fpsyt.2021.723059

**Published:** 2021-08-06

**Authors:** Brittney D. Browning, Melanie L. Schwandt, Mehdi Farokhnia, Sara L. Deschaine, Colin A. Hodgkinson, Lorenzo Leggio

**Affiliations:** ^1^Clinical Psychoneuroendocrinology and Neuropsychopharmacology Section, Translational Addiction Medicine Branch, National Institute on Drug Abuse Intramural Research Program and National Institute on Alcohol Abuse and Alcoholism Division of Intramural Clinical and Biological Research, National Institutes of Health, Baltimore, MD, United States; ^2^Office of the Clinical Director, National Institute on Alcohol Abuse and Alcoholism Division of Intramural Clinical and Biological Research, National Institutes of Health, Bethesda, MD, United States; ^3^Department of Mental Health, Johns Hopkins Bloomberg School of Public Health, Johns Hopkins University, Baltimore, MD, United States; ^4^Laboratory of Neurogenetics, National Institute on Alcohol Abuse and Alcoholism Division of Intramural Clinical and Biological Research, National Institutes of Health, Rockville, MD, United States; ^5^Medication Development Program, National Institute on Drug Abuse Intramural Research Program, National Institutes of Health, Baltimore, MD, United States; ^6^Center for Alcohol and Addiction Studies, Department of Behavioral and Social Sciences, Brown University School of Public Health, Providence, RI, United States; ^7^Division of Addiction Medicine, Department of Medicine, School of Medicine, Johns Hopkins University, Baltimore, MD, United States; ^8^Department of Neuroscience, Georgetown University Medical Center, Washington, DC, United States

**Keywords:** leptin, LEP, LEPR, alcohol, nicotine, anxiety

## Abstract

Comorbidity between alcohol use disorder (AUD) and other addictive and psychiatric disorders is highly prevalent and disabling; however, the underlying biological correlates are not fully understood. Leptin is a peptide hormone known for its role in energy homeostasis and food intake. Furthermore, leptin plays a key role in the activity of the hypothalamic-pituitary-adrenal (HPA) axis and of several neurotransmitter systems that regulate emotionality and behavior. However, human studies that have investigated circulating leptin levels in relation to AUD and affective disorders, such as anxiety and depression, are conflicting. Genetic-based analyses of the leptin gene (*LEP*) and leptin receptor gene (*LEPR*) have the potential of providing more insight into the potential role of the leptin system in AUD and comorbid psychopathology. The aim of the current study was to investigate whether genotypic variations at *LEP* and *LEPR* are associated with measures of alcohol use, nicotine use, anxiety, and depression, all of which represent common comorbidities with AUD. Haplotype association analyses were performed, using data from participants enrolled in screening and natural history protocols at the National Institute on Alcohol Abuse and Alcoholism (NIAAA). Analyses were performed separately in European Americans and African Americans due to the variation in haplotype diversity for most genes between these groups. In the European American group, one *LEP* haplotype (EB2H4) was associated with lower odds of having a current AUD diagnosis, two *LEPR* haplotypes (EB7H3, EB8H3) were associated with lower cigarette pack years and two *LEPR* haplotypes (EB7H2, EB8H2) were associated with higher State-Trait Anxiety Inventory (STAI-T) scores. In the African American group, one *LEP* haplotype (AB2H8) was associated with higher cigarette pack years and one *LEP* haplotype (AB3H2) was associated with lower Fagerström Test for Nicotine Dependence (FTND) scores. Overall, this study found that variations in the leptin and leptin receptor genes are associated with measures of alcohol use, nicotine use, and anxiety. While this preliminary study adds support for a role of the leptin system in AUD and psychopathologies, additional studies are required to fully understand the underlying mechanisms and potential therapeutic implications of these findings.

## Introduction

Alcohol use disorder (AUD) is highly prevalent in the U.S., affecting ~14.1 million adults (1). It is frequently co-morbid with other addictive and psychiatric disorders, and there is substantial evidence suggesting a bidirectional relationship between AUD and other psychopathologies (2). Intricately linked to social relationships, educational attainment, financial status, and general health outcomes, these disorders can lead to poor quality of life for both the affected individual and those close to them (3–5). Despite their high prevalence and comorbidity, the underlying biological correlates of these disorders are not fully understood.

The hormone leptin, a 167-amino acid protein encoded by the leptin gene (*LEP*), is primarily known for its role in regulating food intake and energy expenditure (6). Primarily secreted from white adipose tissue, leptin's effects are mediated through binding to leptin receptors expressed at the cell surface and found throughout the central (CNS) and peripheral nervous systems. In the CNS, highest levels of the leptin receptor gene (*LEPR*) expression are found in hypothalamic nuclei such as the paraventricular nucleus (PVN). This brain region plays a critical role in maintaining homeostasis. Furthermore, the PVN is an important driver of hypothalamic-pituitary-adrenal (HPA) responses (7–9). Abnormalities in HPA axis regulation have been implicated in the pathophysiology of anxiety, depression, and alcohol, nicotine and other substance use disorders (10–15). Furthermore, the HPA-axis-related hormones, cortisol and adrenocorticotropic hormone (ACTH), are inversely correlated with leptin levels (16). In addition to the HPA axis, leptin signals to brain regions involved in the regulation of stress, anxiety, emotion, and behavior, including the hippocampus, amygdala, substantia nigra, nucleus accumbens (NAc), and the ventral tegmental area (VTA) (17–19).

While the role of leptin in addiction remains to be fully confirmed, preclinical and clinical studies suggest that leptin may play a role in addictive behaviors and affective disorders (20, 21). Although there are some inconsistencies between studies, several have linked plasma leptin concentrations to alcohol consumption (22–24) and craving (25–30). Leptin levels have been found to be higher in individuals with alcohol dependence who are sweet preferring vs. those who are sweet averse (31). Additionally, plasma leptin levels have been found to be associated with craving of other substances such as nicotine (32, 33) and cocaine (34). Preclinical genetic studies further support a role of leptin in addictive behaviors; leptin-deficient *ob/ob* mice display reduced locomotor response to amphetamine, and this observation is normalized by leptin administration (19). Leptin signaling has also been shown to regulate cocaine expectancy, cocaine- conditioned reward, and cocaine seeking in rodents (35, 36). In addition to addictive behaviors, *ob/ob* mice exhibit increased anxiety, and treatment with leptin results in a reduction of anxiety-like behaviors (37). Wild-type mice also experienced anxiogenic-like effects after acute leptin administration (38). Interestingly, leptin administration has also been found to alleviate depressive symptoms in relevant animal models, such as tail suspension and forced swim tests (38). Collectively, these studies suggest a role for leptin in multiple psychopathologies.

While a growing body of preclinical research indicates a relationship between leptin signaling and neuropsychiatric disorders, studies in humans are limited and inconsistent, reporting higher, lower, or similar peripheral leptin levels in participants vs. controls (39–43). These inconsistencies could be due to a variety of factors, such as study length, time of day that leptin is measured, metabolic state of the participants, and/or the patient populations heterogeneity [see e.g., (31)]. An alternative approach to identify factors that may account for potential differences is to perform genetic analyses investigating the link between different components of the leptin system and behavioral outcomes. Therefore, we investigated potential associations between haplotype variation at *LEP* and *LEPR*, as well as behavioral phenotypes related to addiction, anxiety, and depression, with the aim of determining whether genetic variation in the leptin system might contribute to observable phenotypic differences in these measures.

## Methods and Materials

### Participants and Setting

Participants included 2,148 individuals screened at the National Institutes of Health (NIH) Intramural Research Program, NIH Clinical Center (Bethesda, Maryland), for participation in research protocols at the National Institute on Alcohol Abuse and Alcoholism (NIAAA). Study participants were screened from 2005 to 2018 under NIAAA screening and natural history protocols (98-AA-0009, 05-AA-0121, 14-AA-0181) approved by the appropriate NIH Institutional Review Board and registered at ClinicalTrials.gov (NCT00001673, NCT00106093, NCT02231840). All participants provided written informed consent for the use of their data, including behavioral and genome data. Demographic characteristics of the participants are summarized in Table 1.

**Table 1:** Descriptive characteristics of the sample.

	**Full Sample**	**European American**	**African American**
	***n* = 2,148**	***n* = 1,177**	***n* = 971**
**Sex**, ***n*****(%)**			
Male	1,381 (64.3%)	747 (63.5%)	634 (65.3%)
Female	767 (35.7%)	430 (36.5%)	337 (34.7%)
**Ethnicity**, ***n*****(%)**			
Hispanic	65 (3.0%)	52 (4.4%)	13 (1.3%)
Not Hispanic	2,057 (95.8%)	1,121 (95.3%)	936 (96.4%)
Unknown/not reported	26 (1.2%)	4 (0.3%)	22 (2.3%)
Age, years, *M* (SD)	39.9 (12.5)	38.5 (13.1)	41.5 (11.6)
Years of education^a^, *M* (SD)	14.4 (3.1)	15.4 (3.0)	13.4 (2.8)
BMI^b^, Kg/m^2^, *M* (SD)	26.7 (5.1)	25.9 (4.6)	27.7 (5.4)
**Current AUD^c^**, ***n*****(%)**			
Yes	1,185 (57.9%)	622 (54.5%)	563 (62.1%)
No	863 (42.1%)	519 (45.5%)	344 (37.9%)

a *Data missing for 256 subjects (155 European American, 101 African American)*.

b *BMI, Body mass index, Data missing for 90 subjects (50 European American, 40 African American)*.

c *AUD, Alcohol Use Disorder, SCID diagnoses were missing for 100 subjects (36 European American, 64 African American)*.

### Genotyping and Single Nucleotide Polymorphism Selection

Genotyping was performed at the NIAAA Laboratory of Neurogenetics on the Illumina OmniExpress BeadChip for the whole genome. The selected candidate gene regions, *LEP* and *LEPR*, were used for this study. Ancestry informative markers (AIMs) were extracted from the Illumina array and ancestral proportions were calculated for all participants (44). For quality control, participants with ≥ 3% of missing genome data were excluded from this study.

Single nucleotide polymorphisms (SNPs) covering regions from 30 Kb upstream to 30 Kb downstream of the target gene [*LEP* (7q32.1) and *LEPR* (1p31.3)] were selected. SNPs with a minor allele frequency of ≤ 0.05 and/or a call rate <90% were excluded from the analysis. Due to differences in haplotype block structure between people from European and African origin for most genes, participants were divided into European American and African American subgroups, based on self-reported race, and separate haplotype analyses were performed in each group. Three *LEPR* SNPs in the African American group violated Hardy-Weinberg Equilibrium but were not removed.

### Haplotype Blocks and Association Analyses

**Figure 1:**
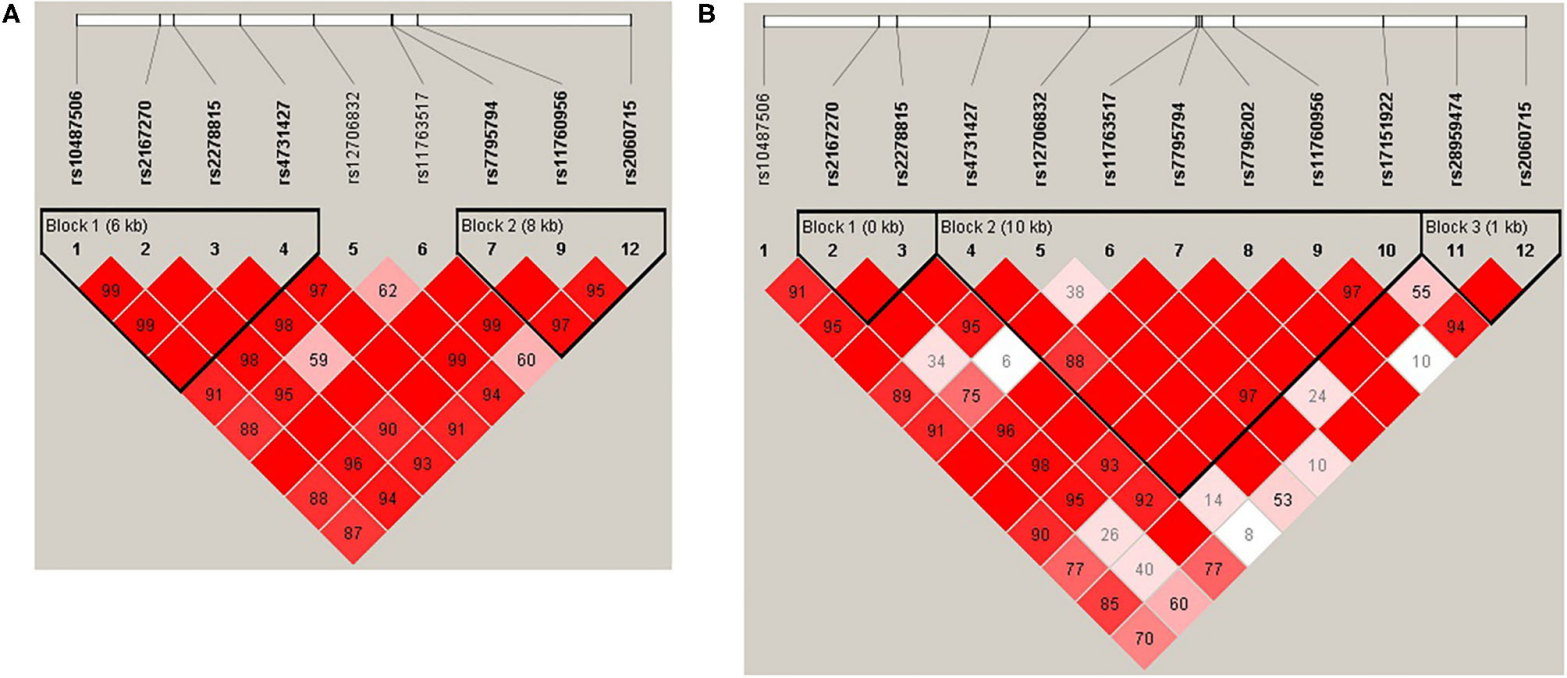
**(A)***LEP* linkage disequilibrium (LD) plot for the European American group. **(B)** *LEP* LD plot for the African American group. Pairwise LD is represented as red for strong LD, blue for non-significant LD, and white for little or no LD.

**Figure 2:**
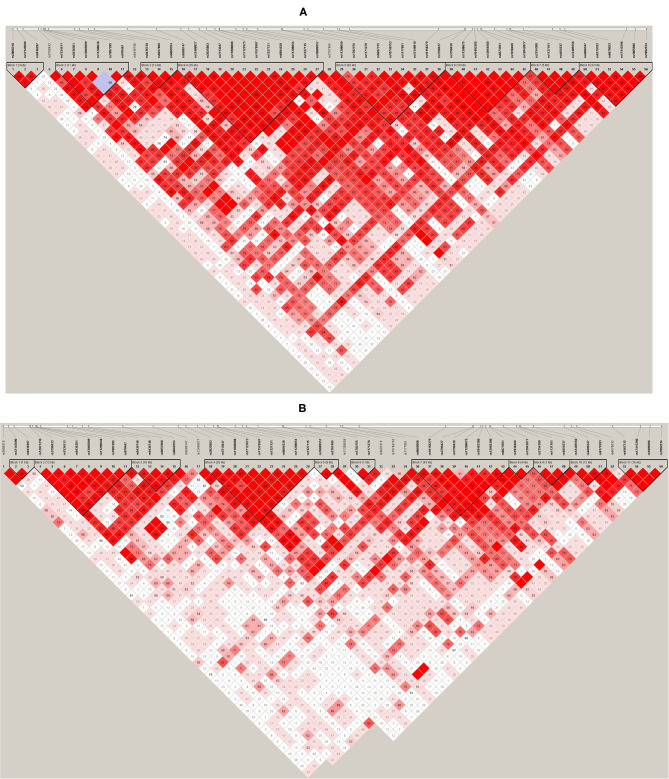
**(A)***LEPR* linkage disequilibrium (LD) plot for the European American group. **(B)** *LEPR* LD plot for the African American group. Pairwise LD is represented as red for strong LD, blue for non-significant LD, and white for little or no LD.

Haploview (45) was used to analyze pair-wise linkage disequilibrium (LD) between all selected SNPs in the regions of *LEP* and *LEPR*. The pair-wise *D*′-values for the SNPs within each region were calculated using Haploview, and haplotype blocks were defined using the default D′/LOD method (45). The LD blocks are presented in Figures 1, 2. Haplotypes were generated separately for European American (*n* = 1,177) and African American (*n* = 971) groups to avoid type I error due to population stratification.

The following assessments were selected for haplotype association analyses: (1) measures of alcohol use: current AUD from Diagnostic and Statistical Manual for Mental Disorders (DSM-IV diagnosis of either alcohol abuse or dependence and DSM-5 of AUD) (46, 47), the Alcohol Use Disorders Identification Test (AUDIT) (48), and number of heavy drinking days (defined as > 4 drinks per day for women and > 5 drink per day for men) and average drinks per drinking day as assessed by the 90-day alcohol Timeline Follow-Back (TLFB) (49); (2) measures of nicotine use: cigarette pack-years (number of cigarette packs smoked per day multiplied by the number of smoking years) and the Fagerström Test for Nicotine Dependence (FTND) (50); (3) a measure of anxiety: the trait scale from the State-Trait Anxiety Inventory (STAI-T) (51); and (4) a measure of depression: the Montgomery-Asberg Depression Rating Scale (MADRS) (52). Sample sizes for the haplotype association analyses vary between assessments due to missing data for each assessment.

Using the defined haplotype blocks, association analyses (linear or logistic regression, depending on the assessment) were then performed in PLINK v1.07 (53, 54) for each gene (LEP and LEPR) and each group (European American and African American). The *p*-values of each association test were corrected for multiple comparisons, using permutation tests (5,000 permutations). All models controlled for age, gender, years of education, and AIMs scores for Europe and Africa. Association analyses were performed for body mass index (BMI), and given no significant associations, BMI was not included as a covariate. A corrected *p*-value of < 0.05 was considered statistically significant.

## Results

### Haplotype Structure and Association Analyses

Haplotype structures differed between the European American and African American groups, as seen in Figures 1, 2. For *LEPR*, there were eight blocks in the European American group and eleven blocks in the African American group. For *LEP*, there were two blocks in the European American group and three blocks in the African American group.

Significant results from the association analyses are displayed in Table 2 and detailed below. Full results can be found in the Supplementary Material. Briefly, a single *LEP* haplotype was associated with lower odds of having a current AUD diagnosis, two *LEPR* haplotypes were associated with lower cigarette pack years and two *LEPR* haplotypes were associated with higher STAI-T scores in the European American group. One *LEP* haplotype was associated with higher cigarette pack years and another *LEP* haplotype was associated with lower FTND scores in the African American group. No significant associations were found with AUDIT scores, number of heavy drinking days, average drinks per drinking day, or MADRS scores.

**Table 2:** Significant results of haplotype association analyses.

**Gene**	**Outcome**	**Group**	**Haplotype**	**Haplotype block**	**SNPs**	**Frequency**	Beta/odds ratio^*^	**STAT**	**Adjusted *p*-value**
LEP	Current AUD diagnosis	European American	GGA, EB2H4	Block 2	rs7795794, rs11760956, rs2060715	0.0413	0.463^*^	9.77	0.0104
LEP	Cigarette pack years	African American	AAAGGGC, AB2H8	Block 2	rs4731427, rs12706832, rs11763517, rs7795794, rs7796202, rs11760956, rs17151922	0.0186	7.26	11.3	0.03959
LEP	FTND score	African American	GG, AB3H2	Block 3	rs28959474, rs2060715	0.205	−0.609	10.2	0.0168
LEPR	Cigarette pack years	European American	AAGC, EB7H3	Block 7	rs2154380, rs1137101, rs4655537, rs12405556	0.201	−2.27	12.7	0.0126
LEPR	Cigarette pack years	European American	ACAAAA, EB8H3	Block 8	rs4606347, rs8179183, rs6678033, rs17415296, rs1805096, rs1892534	0.179	−2.46	13.2	0.0092
LEPR	STAI-T score	European American	GGGA, EB7H2	Block 7	rs2154380, rs1137101, rs4655537, rs12405556	0.263	3.14	10.9	0.03259
LEPR	STAI-T score	European American	GGACAA, EB8H2	Block 8	rs4606347, rs8179183, rs6678033, rs17415296, rs1805096, rs1892534	0.201	3.56	11.8	0.0218

*Full results can be found in the Supplementary Material*.

*LEP, leptin gene; LEPR, leptin receptor gene; SNP, single nucleotide polymorphism; Beta, regression coefficient; STAT, test statistic (t); AUD, alcohol use disorder; FTND, Fagerström Test for Nicotine Dependence; STAI-T, state trait anxiety inventory (trait)*.

* *indicates odds ratio*.

### Leptin Gene

One *LEP* haplotype (EB2H4) was found to be significantly associated with lower odds of having a current AUD diagnosis in the European American group (*n* = 622 cases and 518 controls, *p* < 0.05). This haplotype was formed by rs7795794, rs11760956, and rs2060715.

One *LEP* haplotype (AB2H8) was significantly associated with higher cigarette pack years in the African American group (*n* = 848, *p* < 0.05). This haplotype was formed by rs4731427, rs12706832, rs11763517, rs7795794, rs7796202, rs11760956, and rs17151922. In addition, another *LEP* haplotype (AB3H2) was associated with lower FTND scores in the African American group (*n* = 404, *p* < 0.05). This haplotype was formed by rs28959474 and rs2060715.

### Leptin Receptor Gene

Two *LEPR* haplotypes (EB7H3, EB8H3) were significantly associated with lower cigarette pack years in the European American group (*n* = 1034, *p* < 0.05, *p* < 0.01). Haplotype EB7H3 was formed by rs2154380, rs1137101, rs4655537, and rs12405556; haplotype EB8H3 was formed by rs4606347, rs8179183, rs6678033, rs17415296, rs1805096, and rs1892534.

Two *LEPR* haplotypes (EB7H2, EB8H2) were significantly associated with higher STAI-T scores in the European American group (*n* = 571, all *p's* < 0.05). Haplotype EB7H2 was formed by rs2154380, rs1137101 (Q223R), rs4655537, and rs12405556; and haplotype EB8H2 was formed by rs4606347, rs8179183 (K656N), rs6678033, rs17415296, rs1805096 (P1019P), and rs1892534.

## Discussion

In this study, we examined whether genetic variations at *LEP* and *LEPR* are associated with AUD and measures related to commonly comorbid conditions, specifically cigarette smoking, anxiety, and depression. Our data showed that a single *LEP* haplotype was associated with risk for AUD in the European American group. Whilst no significant associations with AUD were found in the African American group, *LEP* haplotypes were significantly associated with measures of cigarette smoking. *LEPR* haplotypes in the European American group were also significantly associated with measures of cigarette smoking as well as anxiety.

We showed in the present study that AUD diagnosis was associated with genetic variation at the *LEP* gene in a European American sample. The mouse homolog of the *LEP* gene (*Ob*) was first mapped in 1995, and the structure of the human *LEP* gene was later described by Thompson et al., to have three exons separated by two introns (55). In the human gene, exon 1 is separated from exon 2 by a 10 kb intron (intron 1), in which the majority of SNPs in our haplotype blocks were located. We identified a haplotype in *LEP* (EB2H4) associated with lower odds of having a current diagnosis of AUD in the European American sample. The SNPs in this haplotype block were mostly located in intron 1 upstream from exon 2, with one variant (rs2060715) being located downstream from *LEP* in an intergenic region. Previous single SNP association studies have found the same SNP alleles to be associated with lower odds of breast cancer in women, bone mineral density in older men, and to be trending toward association with plasma leptin levels in post-menopausal women (56–58). Interestingly, a haplotype (AB3H2) in the African American sample contained the minor G allele at rs2060715 and was found to be associated with higher FTND score in our study, suggesting the presence of a common functional variant that may broadly contribute to addictive behaviors.

We also identified a *LEP* haplotype in the African American sample associated with nicotine use. In block 2, AB2H8 of the African American sample was associated with higher cigarette pack years. The SNPs in this haplotype were mainly intronic variants located in intron 1 (with the exception of one 3′ UTR variant - rs17151922). Notably, individuals with the CT genotype at rs11763517 were shown to have higher methylation levels at cg00666422, reduced performance in lung expiratory measures, and increased risk of asthma among 18-year-old subjects, particularly women (59). Other studies of these SNP alleles have identified phenotypic differences in systolic blood pressure and breast cancer in women, time to partial antidepressant treatment response, and above average olanzapine-induced weight gain in patients with schizophrenia and schizoaffective disorder (56, 58, 60). Lastly, another study reported reduced pain-induced dopamine release from the striatum and the NAc in the AA *vs*. GG genotype at rs12706832, which may imply decreased salience signaling to negative stimuli (61). Individuals with AA genotype at rs12706832 were additionally found to have lower *LEP* mRNA and protein expression, as well as lower basal and lipopolysaccharide-induced IL-6 levels, compared to individuals homozygous for the minor C allele. Our findings of increased cigarette pack years with this haplotype are interesting given that previous studies have found the individual SNPs in these haplotypes to be associated with lung function, dopamine signaling, and leptin secretion, which renders further investigation into the potential interaction between these identified phenotypes.

In addition to the leptin gene, we also identified haplotypes associated with cigarette use in the leptin receptor gene. The *LEPR* gene has an unusually complicated organization given that the proximal promoter produces two distinct transcripts through alternative splicing, the *LEPR* transcript and leptin receptor overlapping transcript (*LEPROT*, also known as endospanin-1) (62). *LEPROT* overlaps with the 5′ end of *LEPR* and appears to play a role in leptin receptor trafficking, cell surface expression, and leptin resistance. Additionally, the human *LEPR* gene itself can produce 4 isoforms through alternative splicing which vary in their C-terminal length and, thus, ability to activate downstream pathways (63). The haplotypes that we found to be associated with cigarette pack years were located in blocks 7 and 8, which contained SNPs spanning from intron 6 to the 3′ UTR end of *LEPR* (EB7H3 and EB8H3). EB7H3 and EB8H3 were both associated with lower cigarette pack years in the European American group. EB7H3 contains a known coding sequence variant, rs1137101 (A to G transition), that results in a glutamine to arginine substitution (Q223R) at the Corticotropin Releasing Hormone-1 (CRH1) domain of the leptin receptor. While molecular dynamic studies suggest that arginine substitution affects leptin receptor conformation (64), investigations into phenotypic differences resulting from this substitution have produced mixed findings. It is unlikely that our findings are the result of this amino acid substitution, given that another haplotype containing rs1137101 in block 7 (EB7H1) coding for the same amino acid was not associated with cigarette pack years. However, other studies of SNPs in EB7H3 have identified associations with C-reactive protein (CRP) levels and plasma leptin levels in women, soluble leptin receptor levels, E. histolytica infection in children, luminal A breast cancer, tolerance for exercise intensity, and exercise participation (56, 65–70). EB8H3 of *LEPR* also contained a missense variant, rs8179183 (G/C), which results in a lysine to asparagine (K656N) amino acid substitution and a negative to neutral charge change in the membrane-proximal extracellular region of the leptin receptor. Interestingly, in our analysis of cigarette pack years, haplotype EB8H3 was the only haplotype in block 8 containing the minor C allele resulting in the 656N protein, however the functional consequence of this protein, if any, remains unknown. Regarding the other SNPs in this haplotype, functional consequences identified to date include associations with plasma CRP and fibrinogen, osteoporosis, age of menarche, fasting glucose levels, early onset type II diabetes mellitus, and cancer (67, 71–77).

We identified two *LEPR* haplotypes in the European American sample that were associated with anxiety, as indicated by STAI-T score. These haplotypes were located in blocks 7 and 8 and contained SNPs spanning from intron 6 to the 3′ UTR region (EB7H2, EB8H2). Interestingly, we identified haplotypes in blocks 7 and 8 of *LEPR* in the European American sample that were also associated with lower cigarette pack years (EB7H3, EB8H3). Here, it is unlikely that the missense mutations coded for by haplotypes found in blocks 7 (EB7H2; 223R) and 8 (EB8H2; K656) underlie our observed associations with anxiety, as other haplotypes coding for the same amino acid change were not significantly associated with STAI-T score. Previous single SNP association studies have found the SNP alleles in these haplotypes to be associated with plasma leptin and soluble leptin receptor levels, plasma CRP, and other diseases, including breast cancer and diabetes mellitus, suggesting that further evaluation of the potential interaction between these phenotypes is needed. The role of leptin in anxiety remains to be fully understood. One study found no difference in leptin levels between children with anxiety disorders and controls (78). In adult participants, low cerebrospinal fluid (CSF) leptin in females who recently attempted suicide was associated with higher anxiety, while patients who had moderate to severe anxiety had a higher free leptin index, and leptin was positively associated with anxiety in overweight women (43, 79, 80). Our results add to this literature by suggesting that genetic variation at *LEPR* may play a role in anxiety disorders. Lastly, although previous literature has found some relationship between leptin and depression, we found no *LEP* or *LEPR* haplotype associations with MADRS scores.

We report here that genetic variation at *LEP* and *LEPR* was associated with AUD diagnosis, cigarette use, and anxiety. Numerous papers have identified a relationship between leptin and alcohol-related measures [for a recent review, see: (20)], wherein leptin appears to be negatively associated with alcohol craving in current drinkers, and an increase in leptin following alcohol abstinence appears to be positively associated with alcohol craving. Here, we identified only one *LEP* haplotype to be associated with a diagnosis of current AUD in the European American group but found no relationship with TLFB measures of alcohol drinking or AUDIT score. The majority of studies on the relationship between leptin and nicotine typically report the effect of nicotine use on leptin, given that nicotine is known to affect body weight, rather than the effect of leptin on nicotine craving and dependence. However, several studies document a direct relationship between leptin and nicotine craving during abstinence, and another study identified that the magnitude of increase in peripheral leptin, during early phases of withdrawal, is negatively associated with risk of smoking relapse (32, 81, 82). Our findings further suggest a role for the leptin system in nicotine use, as we identified two *LEP* haplotypes to be associated with nicotine dependence and cigarette pack years in the African American group.

Our identified haplotype associations with substance use and anxiety measures, along with findings from previous literature (20, 21, 32, 81–85) suggest that genetic variations within components of the leptin system (peptide/receptor) may be linked to these psychological and behavioral outcomes. These associations raise interesting questions about how anxiety may be linked to leptin and smoking, however, the causative effects of these associations cannot be delineated. Mechanistic studies are needed to understand the biological meaning and underpinnings of these findings. This notion should also be further explored, as the associations observed in this study were not consistent across measures, nor between groups (European American and African American). Additionally, although our sample size was large, replication of these findings in an independent sample and future studies with more comprehensive genetic approaches are imperative, given the limitations of candidate gene analyses, such as the possibility of false positive results. Finally, although this approach does allow for studying a more stable element of the leptin system, it remains unclear how these findings relate to functionality in leptin signaling activation *via* the peptide and/or the receptor, therefore causality cannot be implied.

In summary, our results suggest that *LEP* and *LEPR* are linked to some measures of substance use and anxiety. The mechanisms and casual factors underlying these behaviors include a complex combination of genetic and environmental factors; therefore, much additional work is needed to parse out the potential role of the leptin system in this regard.

## Data Availability Statement

The data analyzed in this study is subject to the following licenses/restrictions: The dataset is managed by and housed within the NIAAA Office of the Clinical Director. Requests to access these datasets should be directed to Melanie L. Schwandt, melanies@mail.nih.gov.

## Ethics Statement

The studies involving human participants were reviewed and approved by NIH Addictions Institutional Review Board. The patients/participants provided their written informed consent to participate in this study.

## Author Contributions

BB: data curation, formal analysis, methodology, and project administration. MS: data curation, formal analysis, investigation, methodology, project administration, resources, and supervision. MF and SD: formal analysis, investigation, and methodology. CH: data curation, formal analysis, investigation, and methodology. LL: conceptualization, investigation, methodology, project administration, and supervision. All authors contributed to the article and approved the submitted version.

## Author Disclaimer

The content of this article is solely the responsibility of the authors and does not necessarily represent the official views of the NIH.

## Conflict of Interest

The authors declare that the research was conducted in the absence of any commercial or financial relationships that could be construed as a potential conflict of interest.

## Publisher's Note

All claims expressed in this article are solely those of the authors and do not necessarily represent those of their affiliated organizations, or those of the publisher, the editors and the reviewers. Any product that may be evaluated in this article, or claim that may be made by its manufacturer, is not guaranteed or endorsed by the publisher.
