# Supplementary material for: Leptin Gene and Leptin Receptor Gene Polymorphisms in Alcohol Use Disorder: Findings Related to Psychopathology
Source: Front Psychiatry. 2021 Aug 6;12:723059. doi: 10.3389/fpsyt.2021.723059 (PMC8377199; doi:10.3389/fpsyt.2021.723059)
Supplement: Supplementary file 1 [file Table_1.DOCX]

**Supplementary Material**

| **LEP and LEPR Haplotype Labels and Frequencies** | | | | | |
| --- | --- | --- | --- | --- | --- |
| **Sample** | **Gene** | **Locus** | **Haplotype** | **Frequency** | **Label** |
| European American | LEP | BLOCK1 | AGAA | 0.4302 | EB1H1 |
| European American | LEP | BLOCK1 | GAGA | 0.3891 | EB1H2 |
| European American | LEP | BLOCK1 | GGAA | 0.09314 | EB1H3 |
| European American | LEP | BLOCK1 | GGGG | 0.0683 | EB1H4 |
| European American | LEP | BLOCK1 | GGGA | 0.0179 | EB1H5 |
| European American | LEP | BLOCK2 | GGG | 0.4948 | EB2H1 |
| European American | LEP | BLOCK2 | GAA | 0.3918 | EB2H2 |
| European American | LEP | BLOCK2 | AGA | 0.06189 | EB2H3 |
| European American | LEP | BLOCK2 | GGA | 0.04134 | EB2H4 |
| European American | LEPR | BLOCK1 | AAA | 0.3949 | EB1H1 |
| European American | LEPR | BLOCK1 | GCA | 0.263 | EB1H2 |
| European American | LEPR | BLOCK1 | ACA | 0.2035 | EB1H3 |
| European American | LEPR | BLOCK1 | AAG | 0.1352 | EB1H4 |
| European American | LEPR | BLOCK2 | GAAAAG | 0.3723 | EB2H1 |
| European American | LEPR | BLOCK2 | AAAAAG | 0.3149 | EB2H2 |
| European American | LEPR | BLOCK2 | GGAAAA | 0.124 | EB2H3 |
| European American | LEPR | BLOCK2 | GGAAGG | 0.09974 | EB2H4 |
| European American | LEPR | BLOCK2 | GAGGAG | 0.05013 | EB2H5 |
| European American | LEPR | BLOCK2 | GGAAAG | 0.03545 | EB2H6 |
| European American | LEPR | BLOCK3 | AGA | 0.3985 | EB3H1 |
| European American | LEPR | BLOCK3 | CAA | 0.3815 | EB3H2 |
| European American | LEPR | BLOCK3 | CGC | 0.1276 | EB3H3 |
| European American | LEPR | BLOCK3 | CGA | 0.08916 | EB3H4 |
| European American | LEPR | BLOCK4 | AGGAGACAGACG | 0.4444 | EB4H1 |
| European American | LEPR | BLOCK4 | GAAGGACGGGAA | 0.2621 | EB4H2 |
| European American | LEPR | BLOCK4 | AGAGAGCAAACG | 0.1488 | EB4H3 |
| European American | LEPR | BLOCK4 | GAAGGACGGGAG | 0.06245 | EB4H4 |
| European American | LEPR | BLOCK4 | AAAGGAAAGAAG | 0.06095 | EB4H5 |
| European American | LEPR | BLOCK5 | ACGAGAAAA | 0.3257 | EB5H1 |
| European American | LEPR | BLOCK5 | ACGGAGACG | 0.2492 | EB5H2 |
| European American | LEPR | BLOCK5 | AAAAAAAAA | 0.1551 | EB5H3 |
| European American | LEPR | BLOCK5 | ACGAGAACA | 0.1247 | EB5H4 |
| European American | LEPR | BLOCK5 | GCGGAAGCA | 0.05826 | EB5H5 |
| European American | LEPR | BLOCK5 | AAAAAAACA | 0.02411 | EB5H6 |
| European American | LEPR | BLOCK5 | GCGGAAACA | 0.02367 | EB5H7 |
| European American | LEPR | BLOCK5 | AAAGAGACG | 0.01077 | EB5H8 |
| European American | LEPR | BLOCK6 | CAAGAAG | 0.3177 | EB6H6 |
| European American | LEPR | BLOCK6 | CCAGGAA | 0.2825 | EB6H7 |
| European American | LEPR | BLOCK6 | AAAAGGG | 0.2107 | EB6H8 |
| European American | LEPR | BLOCK6 | CACGAAG | 0.1882 | EB6H9 |
| European American | LEPR | BLOCK7 | AAAC | 0.3411 | EB7H1 |
| European American | LEPR | BLOCK7 | GGGA | 0.263 | EB7H2 |
| European American | LEPR | BLOCK7 | AAGC | 0.2014 | EB7H3 |
| European American | LEPR | BLOCK7 | GGGC | 0.1946 | EB7H4 |
| European American | LEPR | BLOCK8 | GGGCGG | 0.6045 | EB8H1 |
| European American | LEPR | BLOCK8 | GGACAA | 0.2014 | EB8H2 |
| European American | LEPR | BLOCK8 | ACAAAA | 0.1793 | EB8H3 |
| African American | LEP | BLOCK1 | GG | 0.4561 | AB1H1 |
| African American | LEP | BLOCK1 | AG | 0.386 | AB1H2 |
| African American | LEP | BLOCK1 | GA | 0.1579 | AB1H3 |
| African American | LEP | BLOCK2 | AAAGGGA | 0.2661 | AB2H1 |
| African American | LEP | BLOCK2 | AGAGGGC | 0.1874 | AB2H2 |
| African American | LEP | BLOCK2 | AAGGGAC | 0.1766 | AB2H3 |
| African American | LEP | BLOCK2 | AAAGAGC | 0.1385 | AB2H4 |
| African American | LEP | BLOCK2 | GAAAGGC | 0.1224 | AB2H5 |
| African American | LEP | BLOCK2 | GAAGGGC | 0.04694 | AB2H6 |
| African American | LEP | BLOCK2 | AGGGGGC | 0.02729 | AB2H7 |
| African American | LEP | BLOCK2 | AAAGGGC | 0.01857 | AB2H8 |
| African American | LEP | BLOCK2 | AAAAGGC | 0.01403 | AB2H9 |
| African American | LEP | BLOCK3 | GA | 0.6782 | AB3H1 |
| African American | LEP | BLOCK3 | GG | 0.2051 | AB3H2 |
| African American | LEP | BLOCK3 | AA | 0.1167 | AB3H3 |
| African American | LEPR | BLOCK1 | AA | 0.4406 | AB1H1 |
| African American | LEPR | BLOCK1 | CA | 0.4196 | AB1H2 |
| African American | LEPR | BLOCK1 | AG | 0.1389 | AB1H3 |
| African American | LEPR | BLOCK2 | AAGAGGAG | 0.2852 | AB2H1 |
| African American | LEPR | BLOCK2 | GAGGAAAA | 0.2205 | AB2H2 |
| African American | LEPR | BLOCK2 | GGAAAAAG | 0.1939 | AB2H3 |
| African American | LEPR | BLOCK2 | GAGGAAGG | 0.122 | AB2H4 |
| African American | LEPR | BLOCK2 | GGGAAAAG | 0.07157 | AB2H5 |
| African American | LEPR | BLOCK2 | GAGGAAAG | 0.04861 | AB2H6 |
| African American | LEPR | BLOCK2 | GAGAGGAG | 0.02989 | AB2H7 |
| African American | LEPR | BLOCK2 | GAGAGGAA | 0.01079 | AB2H8 |
| African American | LEPR | BLOCK3 | ACAA | 0.4723 | AB3H1 |
| African American | LEPR | BLOCK3 | CAGA | 0.1883 | AB3H2 |
| African American | LEPR | BLOCK3 | CCGC | 0.1519 | AB3H3 |
| African American | LEPR | BLOCK3 | ACGA | 0.135 | AB3H4 |
| African American | LEPR | BLOCK3 | CCGA | 0.03646 | AB3H5 |
| African American | LEPR | BLOCK4 | AGAGCAAAC | 0.3288 | AB4H1 |
| African American | LEPR | BLOCK4 | AGGACGGGA | 0.2008 | AB4H2 |
| African American | LEPR | BLOCK4 | AGGAAAGAA | 0.1565 | AB4H3 |
| African American | LEPR | BLOCK4 | AGAACAGAC | 0.09731 | AB4H4 |
| African American | LEPR | BLOCK4 | GAGACAGAC | 0.09166 | AB4H5 |
| African American | LEPR | BLOCK4 | AGAACAAAC | 0.07335 | AB4H6 |
| African American | LEPR | BLOCK4 | AGGACAGAA | 0.03089 | AB4H7 |
| African American | LEPR | BLOCK5 | AA | 0.4116 | AB5H1 |
| African American | LEPR | BLOCK5 | GA | 0.2961 | AB5H2 |
| African American | LEPR | BLOCK5 | GG | 0.2915 | AB5H3 |
| African American | LEPR | BLOCK6 | CG | 0.5533 | AB6H1 |
| African American | LEPR | BLOCK6 | AA | 0.4447 | AB6H2 |
| African American | LEPR | BLOCK7 | GAACAAGA | 0.4281 | AB7H1 |
| African American | LEPR | BLOCK7 | GCGCCAGG | 0.1678 | AB7H2 |
| African American | LEPR | BLOCK7 | ACAAAAAG | 0.1091 | AB7H3 |
| African American | LEPR | BLOCK7 | GCAAAAAG | 0.08757 | AB7H4 |
| African American | LEPR | BLOCK7 | GCACCAGG | 0.07268 | AB7H5 |
| African American | LEPR | BLOCK7 | GAACACGA | 0.05518 | AB7H6 |
| African American | LEPR | BLOCK7 | GCAAAAGG | 0.0172 | AB7H7 |
| African American | LEPR | BLOCK7 | GCAACAGG | 0.01551 | AB7H8 |
| African American | LEPR | BLOCK7 | GCACCCGG | 0.01213 | AB7H9 |
| African American | LEPR | BLOCK8 | AG | 0.5 | AB8H1 |
| African American | LEPR | BLOCK8 | AA | 0.274 | AB8H2 |
| African American | LEPR | BLOCK8 | GG | 0.226 | AB8H3 |
| African American | LEPR | BLOCK9 | GGG | 0.5407 | AB9H1 |
| African American | LEPR | BLOCK9 | AAA | 0.4022 | AB9H2 |
| African American | LEPR | BLOCK9 | AAG | 0.05458 | AB9H3 |
| African American | LEPR | BLOCK9 | AAG | 0.05441 | AB9H4 |
| African American | LEPR | BLOCK10 | CGG | 0.6499 | AB10H1 |
| African American | LEPR | BLOCK10 | CAC | 0.1859 | AB10H2 |
| African American | LEPR | BLOCK10 | AGG | 0.1631 | AB10H3 |
| African American | LEPR | BLOCK11 | GCGG | 0.5427 | AB11H1 |
| African American | LEPR | BLOCK11 | GCAA | 0.2577 | AB11H2 |
| African American | LEPR | BLOCK11 | ACAA | 0.1344 | AB11H3 |
| African American | LEPR | BLOCK11 | GAAA | 0.0631 | AB11H4 |
| LEP- leptin gene, LEPR- leptin receptor gene | | | | | |

| ***LEP* Haplotype Association Analysis of Current Alcohol Use Disorder in European American Group** | | | | | | | |
| --- | --- | --- | --- | --- | --- | --- | --- |
| **Block** | **SNPs** | **Haplotype** | **Frequency** | **Odds Ratio** | **STAT** | ***p*-value** | **Adjusted *p*-value** |
| 1 | rs10487506, rs2167270, rs2278815, rs4731427 | GGGG | 0.0683 | 0.917 | 0.232 | 0.63 | 0.996 |
|  |  | GAGA | 0.389 | 1.2 | 3.83 | 0.0504 | 0.2675 |
|  |  | GGGA | 0.0179 | 0.486 | 4.14 | 0.0419 | 0.23 |
|  |  | AGAA | 0.43 | 0.89 | 1.63 | 0.201 | 0.7109 |
|  |  | GGAA | 0.0931 | 1.03 | 0.032 | 0.858 | 1 |
| 2 | rs7795794, rs11760956, rs2060715 | GAA | 0.392 | 1.23 | 4.69 | 0.0303 | 0.1692 |
|  |  | AGA | 0.0619 | 0.969 | 0.0275 | 0.868 | 1 |
|  |  | GGA | 0.0413 | 0.463 | 9.77 | 0.0018 | **0.0104** |
|  |  | GGG | 0.495 | 0.901 | 1.26 | 0.261 | 0.813 |
| *n*=622 cases, 518 controls and 37 missing  Abbreviations: LEP- leptin gene, SNP- single nucleotide polymorphism, STAT- test statistic  Adjusted *p*-values in bold indicate statistically significant associations | | | | | | | |

| ***LEP* Haplotype Association Analysis of Current Alcohol Use Disorder in African American Group** | | | | | | | |
| --- | --- | --- | --- | --- | --- | --- | --- |
| **Block** | **SNPs** | **Haplotype** | **Frequency** | **Odds ratio** | **STAT** | ***p*-value** | **Adjusted *p*-value** |
| 1 | rs2167270, rs2278815 | GA | 0.158 | 1.11 | 0.532 | 0.466 | 0.9992 |
|  |  | AG | 0.386 | 0.991 | 0.00723 | 0.932 | 1 |
|  |  | GG | 0.456 | 0.956 | 0.186 | 0.667 | 1 |
| 2 | rs4731427, rs12706832, rs11763517, rs7795794, rs7796202, rs11760956, rs17151922 | AAAGGGA | 0.266 | 1.07 | 0.355 | 0.552 | 1 |
|  |  | AAAGAGC | 0.139 | 0.881 | 0.757 | 0.384 | 0.994 |
|  |  | GAAAGGC | 0.122 | 0.992 | 0.0026 | 0.959 | 1 |
|  |  | AGAGGGC | 0.187 | 0.899 | 0.661 | 0.416 | 0.9968 |
|  |  | AAGGGAC | 0.177 | 1.04 | 0.104 | 0.747 | 1 |
|  |  | AAAGGGC | 0.0186 | 2.69 | 4.29 | 0.0382 | 0.3749 |
|  |  | GAAGGGC | 0.0469 | 0.732 | 1.59 | 0.208 | 0.9276 |
|  |  | AAAAGGC | 0.014 | 2.21 | 2.1 | 0.147 | 0.8368 |
|  |  | AGGGGGC | 0.0273 | 1.42 | 0.96 | 0.327 | 0.9866 |
| 3 | rs28959474, rs2060715 | GG | 0.205 | 1.18 | 1.67 | 0.196 | 0.914 |
|  |  | AA | 0.117 | 0.97 | 0.0364 | 0.849 | 1 |
|  |  | GA | 0.678 | 0.9 | 0.933 | 0.334 | 0.9886 |
| *n*=562 cases, 344 controls, and 65 missing  Abbreviations: LEP- leptin gene, SNP- single nucleotide polymorphism, STAT- test statistic | | | | | | | |

| ***LEP* Haplotype Association Analysis of Heavy Drinking Days in European American Group** | | | | | | | |
| --- | --- | --- | --- | --- | --- | --- | --- |
| **Block** | **SNPs** | **Haplotype** | **Frequency** | **BETA** | **STAT** | ***p*-value** | **Adjusted *p*-value** |
| 1 | rs10487506, rs2167270, rs2278815, rs4731427 | GGGG | 0.0683 | 2.76 | 0.966 | 0.326 | 0.887 |
|  |  | GAGA | 0.389 | 0.452 | 0.0946 | 0.758 | 0.9996 |
|  |  | GGGA | 0.0179 | -3.54 | 0.369 | 0.544 | 0.9872 |
|  |  | AGAA | 0.43 | -1.14 | 0.63 | 0.428 | 0.9546 |
|  |  | GGAA | 0.0931 | 0.204 | 0.00693 | 0.934 | 1 |
| 2 | rs7795794, rs11760956, rs2060715 | GAA | 0.392 | 0.762 | 0.266 | 0.606 | 0.9952 |
|  |  | AGA | 0.0619 | 2.67 | 0.829 | 0.363 | 0.9186 |
|  |  | GGA | 0.0413 | -5.15 | 1.66 | 0.198 | 0.7127 |
|  |  | GGG | 0.495 | -1.45 | 0.993 | 0.319 | 0.8806 |
| *n*=1037  Abbreviations: LEP- leptin gene, SNP- single nucleotide polymorphism, STAT- test statistic, BETA- regression coefficient | | | | | | | |

| ***LEP* Haplotype Association Analysis of Heavy Drinking Days in African American Group** | | | | | | | |
| --- | --- | --- | --- | --- | --- | --- | --- |
| **Block** | **SNPs** | **Haplotype** | **Frequency** | **BETA** | **STAT** | ***p*-value** | **Adjusted *p*-value** |
| 1 | rs2167270, rs2278815 | GA | 0.158 | 1.81 | 0.564 | 0.453 | 0.9978 |
|  |  | AG | 0.386 | 1.96 | 1.27 | 0.259 | 0.9612 |
|  |  | GG | 0.456 | -2.89 | 2.78 | 0.096 | 0.6875 |
| 2 | rs4731427, rs12706832, rs11763517, rs7795794, rs7796202, rs11760956, rs17151922 | AAAGGGA | 0.266 | -1.18 | 0.385 | 0.535 | 0.9998 |
|  |  | AAAGAGC | 0.139 | -3.18 | 1.65 | 0.199 | 0.9122 |
|  |  | GAAAGGC | 0.122 | -2.03 | 0.582 | 0.446 | 0.9976 |
|  |  | AGAGGGC | 0.187 | 0.596 | 0.0753 | 0.784 | 1 |
|  |  | AAGGGAC | 0.177 | 3.07 | 1.86 | 0.173 | 0.8774 |
|  |  | AAAGGGC | 0.0186 | 11.3 | 3.17 | 0.0751 | 0.5983 |
|  |  | GAAGGGC | 0.0469 | -4.46 | 1.18 | 0.277 | 0.9696 |
|  |  | AAAAGGC | 0.014 | 13.8 | 2.93 | 0.0875 | 0.6527 |
|  |  | AGGGGGC | 0.0273 | 8.09 | 2.19 | 0.14 | 0.8162 |
| 3 | rs28959474, rs2060715 | GG | 0.205 | 3.23 | 2.36 | 0.125 | 0.7806 |
|  |  | AA | 0.117 | -3.19 | 1.46 | 0.227 | 0.9386 |
|  |  | GA | 0.678 | -0.88 | 0.238 | 0.626 | 1 |
| *n*=854  Abbreviations: LEP- leptin gene, SNP- single nucleotide polymorphism, STAT- test statistic, BETA- regression coefficient | | | | | | | |

| ***LEP* Haplotype Association Analysis of Average Drinks/Drinking Day in European American Group** | | | | | | | |
| --- | --- | --- | --- | --- | --- | --- | --- |
| **Block** | **SNPs** | **Haplotype** | **Frequency** | **BETA** | **STAT** | ***p*-value** | **Adjusted *p*-value** |
| 1 | rs10487506, rs2167270, rs2278815, rs4731427 | GGGG | 0.0683 | 0.401 | 0.343 | 0.558 | 0.9876 |
|  |  | GAGA | 0.389 | -0.106 | 0.0871 | 0.768 | 0.9996 |
|  |  | GGGA | 0.0179 | -0.689 | 0.237 | 0.627 | 0.9956 |
|  |  | AGAA | 0.43 | -0.0976 | 0.0775 | 0.781 | 0.9996 |
|  |  | GGAA | 0.0931 | 0.254 | 0.18 | 0.671 | 0.998 |
| 2 | rs7795794, rs11760956, rs2060715 | GAA | 0.392 | 0.135 | 0.141 | 0.707 | 0.9988 |
|  |  | AGA | 0.0619 | 0.567 | 0.631 | 0.427 | 0.9514 |
|  |  | GGA | 0.0413 | -1.85 | 3.64 | 0.0565 | 0.2899 |
|  |  | GGG | 0.495 | -0.131 | 0.135 | 0.713 | 0.999 |
| *n*=1032 | | | | | | | |

Abbreviations: LEP- leptin gene, SNP- single nucleotide polymorphism, STAT- test statistic, BETA- regression coefficient

| ***LEP* Haplotype Association Analysis of Average Drinks/Drinking Day in African American Group** | | | | | | | |
| --- | --- | --- | --- | --- | --- | --- | --- |
| **Block** | **SNPs** | **Haplotype** | **Frequency** | **BETA** | **STAT** | ***p*-value** | **Adjusted *p*-value** |
| 1 | rs2167270, rs2278815 | GA | 0.158 | 0.252 | 0.194 | 0.66 | 1 |
|  |  | AG | 0.386 | 0.22 | 0.287 | 0.592 | 1 |
|  |  | GG | 0.456 | -0.348 | 0.722 | 0.396 | 0.9958 |
| 2 | rs4731427, rs12706832, rs11763517, rs7795794, rs7796202, rs11760956, rs17151922 | AAAGGGA | 0.266 | -0.331 | 0.539 | 0.463 | 0.999 |
|  |  | AAAGAGC | 0.139 | -0.643 | 1.21 | 0.271 | 0.9704 |
|  |  | GAAAGGC | 0.122 | 0.296 | 0.221 | 0.639 | 1 |
|  |  | AGAGGGC | 0.187 | 0.183 | 0.128 | 0.721 | 1 |
|  |  | AAGGGAC | 0.177 | 0.242 | 0.207 | 0.649 | 1 |
|  |  | AAAGGGC | 0.0186 | 0.772 | 0.269 | 0.604 | 1 |
|  |  | GAAGGGC | 0.0469 | 0.267 | 0.0738 | 0.786 | 1 |
|  |  | AAAAGGC | 0.014 | 1.07 | 0.317 | 0.574 | 0.9998 |
|  |  | AGGGGGC | 0.0273 | 0.752 | 0.34 | 0.56 | 0.9998 |
| 3 | rs28959474, rs2060715 | GG | 0.205 | 0.813 | 2.66 | 0.103 | 0.7101 |
|  |  | AA | 0.117 | -0.424 | 0.464 | 0.496 | 0.9996 |
|  |  | GA | 0.678 | -0.395 | 0.856 | 0.355 | 0.9902 |
| *n*=847  Abbreviations: LEP- leptin gene, SNP- single nucleotide polymorphism, STAT- test statistic, BETA- regression coefficient | | | | | | | |

| ***LEP* Haplotype Association Analysis of Alcohol Use Disorders Identification Test Score in European American Group** | | | | | | | |
| --- | --- | --- | --- | --- | --- | --- | --- |
| **Block** | **SNPs** | **Haplotype** | **Frequency** | **BETA** | **STAT** | ***p*-value** | **Adjusted *p*-value** |
| 1 | rs10487506, rs2167270, rs2278815, rs4731427 | GGGG | 0.0683 | 0.286 | 0.0634 | 0.801 | 1 |
|  |  | GAGA | 0.389 | 0.451 | 0.619 | 0.432 | 0.9592 |
|  |  | GGGA | 0.0179 | -0.999 | 0.252 | 0.616 | 0.9962 |
|  |  | AGAA | 0.43 | -0.24 | 0.184 | 0.668 | 0.9986 |
|  |  | GGAA | 0.0931 | -0.566 | 0.336 | 0.563 | 0.9894 |
| 2 | rs7795794, rs11760956, rs2060715 | GAA | 0.392 | 0.823 | 2.08 | 0.15 | 0.6125 |
|  |  | AGA | 0.0619 | 0.225 | 0.0347 | 0.852 | 1 |
|  |  | GGA | 0.0413 | -1.11 | 0.635 | 0.426 | 0.9562 |
|  |  | GGG | 0.495 | -0.663 | 1.38 | 0.241 | 0.783 |
| *n*=692  Abbreviations: LEP- leptin gene, SNP- single nucleotide polymorphism, STAT- test statistic, BETA- regression coefficient | | | | | | | |

| ***LEP* Haplotype Association Analysis of Alcohol Use Disorders Identification Test Score in African American Group** | | | | | | | |
| --- | --- | --- | --- | --- | --- | --- | --- |
| **Block** | **SNPs** | **Haplotype** | **Frequency** | **BETA** | **STAT** | ***p*-value** | **Adjusted *p*-value** |
| 1 | rs2167270, rs2278815 | GA | 0.158 | 0.235 | 0.0744 | 0.785 | 1 |
|  |  | AG | 0.386 | 1.05 | 3.05 | 0.0811 | 0.6265 |
|  |  | GG | 0.456 | -1.14 | 3.68 | 0.0556 | 0.5009 |
| 2 | rs4731427, rs12706832, rs11763517, rs7795794, rs7796202, rs11760956, rs17151922 | AAAGGGA | 0.266 | 0.924 | 2.01 | 0.156 | 0.8542 |
|  |  | AAAGAGC | 0.139 | -1.61 | 3.62 | 0.0575 | 0.5089 |
|  |  | GAAAGGC | 0.122 | -1.41 | 2.39 | 0.123 | 0.7746 |
|  |  | AGAGGGC | 0.187 | 0.145 | 0.0352 | 0.851 | 1 |
|  |  | AAGGGAC | 0.177 | 0.376 | 0.246 | 0.62 | 1 |
|  |  | AAAGGGC | 0.0186 | 2.96 | 1.9 | 0.169 | 0.8766 |
|  |  | GAAGGGC | 0.0469 | -0.495 | 0.135 | 0.713 | 1 |
|  |  | AAAAGGC | 0.014 | 2.11 | 0.607 | 0.436 | 0.9978 |
|  |  | AGGGGGC | 0.0273 | 1.76 | 0.881 | 0.348 | 0.9898 |
| 3 | rs28959474, rs2060715 | GG | 0.205 | 0.615 | 0.689 | 0.407 | 0.9964 |
|  |  | AA | 0.117 | -0.0052 | 3.40E-05 | 0.995 | 1 |
|  |  | GA | 0.678 | -0.422 | 0.471 | 0.493 | 0.9994 |
| *n*=631  Abbreviations: LEP- leptin gene, SNP- single nucleotide polymorphism, STAT- test statistic, BETA- regression coefficient | | | | | | | |

| ***LEP* Haplotype Association Analysis of Cigarette Pack Years in European American Group** | | | | | | | |
| --- | --- | --- | --- | --- | --- | --- | --- |
| **Block** | **SNPs** | **Haplotype** | **Frequency** | **BETA** | **STAT** | ***p*-value** | **Adjusted *p*-value** |
| 1 | rs10487506, rs2167270, rs2278815, rs4731427 | GGGG | 0.0683 | -0.876 | 0.731 | 0.393 | 0.935 |
|  |  | GAGA | 0.389 | -0.143 | 0.0704 | 0.791 | 1 |
|  |  | GGGA | 0.0179 | -1.41 | 0.448 | 0.503 | 0.9792 |
|  |  | AGAA | 0.43 | 0.833 | 2.51 | 0.114 | 0.4949 |
|  |  | GGAA | 0.0931 | -1.09 | 1.52 | 0.219 | 0.7469 |
| 2 | rs7795794, rs11760956, rs2060715 | GAA | 0.392 | -0.0595 | 0.0121 | 0.912 | 1 |
|  |  | AGA | 0.0619 | -0.943 | 0.774 | 0.379 | 0.9262 |
|  |  | GGA | 0.0413 | -2.02 | 1.96 | 0.162 | 0.6319 |
|  |  | GGG | 0.495 | 0.485 | 0.834 | 0.361 | 0.9142 |
| *n*=1034  Abbreviations: LEP- leptin gene, SNP- single nucleotide polymorphism, STAT- test statistic, BETA- regression coefficient | | | | | | | |

| ***LEP* Haplotype Association Analysis of Cigarette Pack Years in African American Group** | | | | | | | |
| --- | --- | --- | --- | --- | --- | --- | --- |
| **Block** | **SNPs** | **Haplotype** | **Frequency** | **BETA** | **STAT** | ***p*-value** | **Adjusted *p*-value** |
| 1 | rs2167270, rs2278815 | GA | 0.158 | -1.97 | 5.8 | 0.0162 | 0.2238 |
|  |  | AG | 0.386 | -0.021 | 0.00127 | 0.972 | 1 |
|  |  | GG | 0.456 | 1.02 | 3.05 | 0.0809 | 0.6229 |
| 2 | rs4731427, rs12706832, rs11763517, rs7795794, rs7796202, rs11760956, rs17151922 | AAAGGGA | 0.266 | 0.995 | 2.36 | 0.125 | 0.777 |
|  |  | AAAGAGC | 0.139 | 0.261 | 0.098 | 0.754 | 1 |
|  |  | GAAAGGC | 0.122 | -0.378 | 0.178 | 0.673 | 1 |
|  |  | AGAGGGC | 0.187 | -1.81 | 6.06 | 0.0141 | 0.2012 |
|  |  | AAGGGAC | 0.177 | -0.166 | 0.0478 | 0.827 | 1 |
|  |  | AAAGGGC | 0.0186 | 7.26 | 11.3 | 0.000807 | **0.03959** |
|  |  | GAAGGGC | 0.0469 | -0.595 | 0.185 | 0.667 | 1 |
|  |  | AAAAGGC | 0.014 | 2.46 | 0.934 | 0.334 | 0.9884 |
|  |  | AGGGGGC | 0.0273 | -1.49 | 0.659 | 0.417 | 0.9968 |
| 3 | rs28959474, rs2060715 | GG | 0.205 | -1.55 | 4.77 | 0.0292 | 0.3243 |
|  |  | AA | 0.117 | 0.373 | 0.169 | 0.681 | 1 |
|  |  | GA | 0.678 | 0.981 | 2.56 | 0.11 | 0.7357 |
| *n*=848  Abbreviations: LEP- leptin gene, SNP- single nucleotide polymorphism, STAT- test statistic, BETA- regression coefficient  Adjusted *p*-values in bold indicate statistically significant associations | | | | | | | |

| ***LEP* Haplotype Association Analysis of Fagerstr****öm Test for Nicotine Dependence Score in European American Group** | | | | | | | |
| --- | --- | --- | --- | --- | --- | --- | --- |
| **Block** | **SNPs** | **Haplotype** | **Frequency** | **BETA** | **STAT** | ***p*-value** | **Adjusted *p*-value** |
| 1 | rs10487506, rs2167270, rs2278815, rs4731427 | GGGG | 0.0683 | 0.0549 | 0.0243 | 0.876 | 1 |
|  |  | GAGA | 0.389 | -0.302 | 2.76 | 0.0976 | 0.4769 |
|  |  | GGGA | 0.0179 | 0.632 | 0.558 | 0.455 | 0.9754 |
|  |  | AGAA | 0.43 | 0.301 | 2.81 | 0.0946 | 0.4647 |
|  |  | GGAA | 0.0931 | -0.05 | 0.0293 | 0.864 | 1 |
| 2 | rs7795794, rs11760956, rs2060715 | GAA | 0.392 | -0.27 | 2.28 | 0.131 | 0.5783 |
|  |  | AGA | 0.0619 | -0.036 | 0.0094 | 0.923 | 1 |
|  |  | GGA | 0.0413 | 0.0917 | 0.0267 | 0.87 | 1 |
|  |  | GGG | 0.495 | 0.252 | 2.03 | 0.155 | 0.6427 |
| *n*=397, Fagerström Test for Nicotine Dependence score is only calculated for smokers  Abbreviations: LEP- leptin gene, SNP- single nucleotide polymorphism, STAT- test statistic, BETA- regression coefficient | | | | | | | |

| ***LEP* Haplotype Association Analysis of Fagerström Test for Nicotine Dependence Score in African American Group** | | | | | | | |
| --- | --- | --- | --- | --- | --- | --- | --- |
| **Block** | **SNPs** | **Haplotype** | **Frequency** | **BETA** | **STAT** | ***p*-value** | **Adjusted *p*-value** |
| 1 | rs2167270, rs2278815 | GA | 0.158 | -0.499 | 5.31 | 0.0217 | 0.2398 |
|  |  | AG | 0.386 | -0.111 | 0.414 | 0.52 | 0.9994 |
|  |  | GG | 0.456 | 0.405 | 5.85 | 0.016 | 0.1848 |
| 2 | rs4731427, rs12706832, rs11763517, rs7795794, rs7796202, rs11760956, rs17151922 | AAAGGGA | 0.266 | 0.192 | 1.14 | 0.286 | 0.9786 |
|  |  | AAAGAGC | 0.139 | 0.492 | 3.76 | 0.0531 | 0.4845 |
|  |  | GAAAGGC | 0.122 | -0.261 | 0.946 | 0.331 | 0.9898 |
|  |  | AGAGGGC | 0.187 | -0.397 | 3.73 | 0.0543 | 0.4913 |
|  |  | AAGGGAC | 0.177 | -0.144 | 0.427 | 0.514 | 0.9992 |
|  |  | AAAGGGC | 0.0186 | 0.583 | 1.22 | 0.27 | 0.9732 |
|  |  | GAAGGGC | 0.0469 | 0.144 | 0.123 | 0.726 | 1 |
|  |  | AAAAGGC | 0.014 | 0.138 | 0.0407 | 0.84 | 1 |
|  |  | AGGGGGC | 0.0273 | -0.16 | 0.12 | 0.73 | 1 |
| 3 | rs28959474, rs2060715 | GG | 0.205 | -0.609 | 10.2 | 0.00148 | **0.0168** |
|  |  | AA | 0.117 | 0.226 | 0.729 | 0.394 | 0.9962 |
|  |  | GA | 0.678 | 0.402 | 5.37 | 0.021 | 0.2344 |
| *n*=404, Fagerström Test for Nicotine Dependence score is only calculated for smokers  Abbreviations: LEP- leptin gene, SNP- single nucleotide polymorphism, STAT- test statistic, BETA- regression coefficient  Adjusted *p*-values in bold indicate statistically significant associations | | | | | | | |

| ***LEP* Haplotype Association Analysis of State-Trait Anxiety Inventory Trait Score in European American Group** | | | | | | | |
| --- | --- | --- | --- | --- | --- | --- | --- |
| **Block** | **SNPs** | **Haplotype** | **Frequency** | **BETA** | **STAT** | ***p*-value** | **Adjusted *p*-value** |
| 1 | rs10487506, rs2167270, rs2278815, rs4731427 | GGGG | 0.0683 | 2.81 | 2.81 | 0.0945 | 0.4503 |
|  |  | GAGA | 0.389 | -0.411 | 0.244 | 0.621 | 0.9948 |
|  |  | GGGA | 0.0179 | -4.69 | 2.34 | 0.127 | 0.5549 |
|  |  | AGAA | 0.43 | -0.183 | 0.0482 | 0.826 | 1 |
|  |  | GGAA | 0.0931 | 0.602 | 0.188 | 0.665 | 0.9978 |
| 2 | rs7795794, rs11760956, rs2060715 | GAA | 0.392 | 0.082 | 0.00969 | 0.922 | 1 |
|  |  | AGA | 0.0619 | 2.84 | 2.56 | 0.11 | 0.5017 |
|  |  | GGA | 0.0413 | -3.74 | 3.26 | 0.0714 | 0.3705 |
|  |  | GGG | 0.495 | -0.109 | 0.0171 | 0.896 | 1 |
| *n*=571  Abbreviations: LEP- leptin gene, SNP- single nucleotide polymorphism, STAT- test statistic, BETA- regression coefficient | | | | | | | |

| ***LEP* Haplotype Association Analysis of State-Trait Anxiety Inventory Trait Score in African American Group** | | | | | | | |
| --- | --- | --- | --- | --- | --- | --- | --- |
| **Block** | **SNPs** | **Haplotype** | **Frequency** | **BETA** | **STAT** | ***p*-value** | **Adjusted p-value** |
| 1 | rs2167270, rs2278815 | GA | 0.158 | 1.39 | 2.13 | 0.145 | 0.834 |
|  |  | AG | 0.386 | 0.429 | 0.378 | 0.539 | 1 |
|  |  | GG | 0.456 | -1.18 | 2.85 | 0.0921 | 0.6789 |
| 2 | rs4731427, rs12706832, rs11763517, rs7795794, rs7796202, rs11760956, rs17151922 | AAAGGGA | 0.266 | 0.554 | 0.513 | 0.474 | 0.9996 |
|  |  | AAAGAGC | 0.139 | -2.53 | 6.35 | 0.012 | 0.1446 |
|  |  | GAAAGGC | 0.122 | -1.3 | 1.46 | 0.227 | 0.9466 |
|  |  | AGAGGGC | 0.187 | 0.417 | 0.232 | 0.63 | 1 |
|  |  | AAGGGAC | 0.177 | 0.231 | 0.0698 | 0.792 | 1 |
|  |  | AAAGGGC | 0.0186 | 3.27 | 1.81 | 0.18 | 0.9 |
|  |  | GAAGGGC | 0.0469 | 0.87 | 0.303 | 0.582 | 1 |
|  |  | AAAAGGC | 0.014 | 3.39 | 1.07 | 0.302 | 0.985 |
|  |  | AGGGGGC | 0.0273 | 3.85 | 3.3 | 0.0695 | 0.5741 |
| 3 | rs28959474, rs2060715 | GG | 0.205 | 2.06 | 6.09 | 0.0138 | 0.1648 |
|  |  | AA | 0.117 | -0.6 | 0.327 | 0.568 | 1 |
|  |  | GA | 0.678 | -1.2 | 2.87 | 0.0909 | 0.6743 |
| *n*=642  Abbreviations: LEP- leptin gene, SNP- single nucleotide polymorphism, STAT- test statistic, BETA- regression coefficient | | | | | | | |

| ***LEP* Haplotype Association Analysis of Montgomery-Asberg Depression Rating Scale Score in European American Group** | | | | | | | |
| --- | --- | --- | --- | --- | --- | --- | --- |
| **Block** | **SNPs** | **Haplotype** | **Frequency** | **BETA** | **STAT** | ***p*-value** | **Adjusted *p*-value** |
| 1 | rs1048750, rs2167270, rs2278815, rs4731427 | GGGG | 0.0683 | 0.5 | 0.27 | 0.604 | 0.9948 |
|  |  | GAGA | 0.389 | -0.105 | 0.0432 | 0.835 | 1 |
|  |  | GGGA | 0.0179 | -1.36 | 0.434 | 0.51 | 0.9818 |
|  |  | AGAA | 0.43 | -0.071 | 0.0204 | 0.886 | 1 |
|  |  | GGAA | 0.0931 | 0.364 | 0.176 | 0.675 | 0.9984 |
| 2 | rs7795794, rs11760956, rs2060715 | GAA | 0.392 | -0.144 | 0.0799 | 0.778 | 0.9998 |
|  |  | AGA | 0.0619 | 0.763 | 0.581 | 0.446 | 0.9658 |
|  |  | GGA | 0.0413 | -2.7 | 3.6 | 0.058 | 0.2971 |
|  |  | GGG | 0.495 | 0.163 | 0.104 | 0.748 | 0.9998 |
| *n*=830  Abbreviations: LEP- leptin gene, SNP- single nucleotide polymorphism, STAT- test statistic, BETA- regression coefficient | | | | | | | |

| ***LEP* Haplotype Association Analysis of Montgomery-Asberg Depression Rating Scale Score in African American Group** | | | | | | | |
| --- | --- | --- | --- | --- | --- | --- | --- |
| **Block** | **SNPs** | **Haplotype** | **Frequency** | **BETA** | **STAT** | ***p*-value** | **Adjusted *p*-value** |
| 1 | rs2167270, rs2278815 | GA | 0.158 | 0.279 | 0.208 | 0.648 | 1 |
|  |  | AG | 0.386 | 0.0175 | 0.00158 | 0.968 | 1 |
|  |  | GG | 0.456 | -0.16 | 0.134 | 0.714 | 1 |
| 2 | rs4731427, rs12706832, rs11763517, rs7795794, rs7796202, rs11760956, rs17151922 | AAAGGGA | 0.266 | 0.228 | 0.212 | 0.645 | 1 |
|  |  | AAAGAGC | 0.139 | -1.21 | 3.73 | 0.0538 | 0.4783 |
|  |  | GAAAGGC | 0.122 | -0.093 | 0.0192 | 0.89 | 1 |
|  |  | AGAGGGC | 0.187 | 0.267 | 0.235 | 0.628 | 1 |
|  |  | AAGGGAC | 0.177 | -0.33 | 0.341 | 0.559 | 0.9998 |
|  |  | AAAGGGC | 0.0186 | 0.0997 | 0.00409 | 0.949 | 1 |
|  |  | GAAGGGC | 0.0469 | 0.939 | 0.819 | 0.366 | 0.994 |
|  |  | AAAAGGC | 0.014 | 4.31 | 5.29 | 0.0217 | 0.2362 |
|  |  | AGGGGGC | 0.0273 | 0.91 | 0.476 | 0.49 | 0.9982 |
| 3 | rs28959474, rs2060715 | GG | 0.205 | 0.941 | 3.18 | 0.0749 | 0.6015 |
|  |  | AA | 0.117 | -0.0523 | 0.00601 | 0.938 | 1 |
|  |  | GA | 0.678 | -0.68 | 2.22 | 0.137 | 0.8098 |
| *n*=815  Abbreviations: LEP- leptin gene, SNP- single nucleotide polymorphism, STAT- test statistic, BETA- regression coefficient | | | | | | | |

| ***LEP* Haplotype Association Analysis of Body Mass Index in European American Group** | | | | | | | |
| --- | --- | --- | --- | --- | --- | --- | --- |
| **Block** | **SNPs** | **Haplotype** | **Frequency** | **BETA** | **STAT** | ***p*-value** | **Adjusted *p*-value** |
| 1 | rs10487506, rs2167270, rs2278815, rs4731427 | GGGG | 0.0683 | 0.666 | 3.17 | 0.0753 | 0.3757 |
|  |  | GAGA | 0.389 | -0.357 | 3.46 | 0.0632 | 0.3209 |
|  |  | GGGA | 0.0179 | 0.372 | 0.261 | 0.61 | 0.9956 |
|  |  | AGAA | 0.43 | 0.135 | 0.515 | 0.473 | 0.9698 |
|  |  | GGAA | 0.0931 | 0.0229 | 0.0051 | 0.943 | 1 |
| 2 | rs7795794, rs11760956, rs2060715 | GAA | 0.392 | -0.495 | 6.6 | 0.0103 | 0.06439 |
|  |  | AGA | 0.0619 | 0.558 | 2.02 | 0.155 | 0.6145 |
|  |  | GGA | 0.0413 | 1.07 | 4.63 | 0.0316 | 0.1814 |
|  |  | GGG | 0.495 | 0.207 | 1.18 | 0.278 | 0.8382 |
| *n*=1127  Abbreviations: LEP- leptin gene, SNP- single nucleotide polymorphism, STAT- test statistic, BETA- regression coefficient | | | | | | | |

| ***LEP* Haplotype Association Analysis of Body Mass Index in African American Group** | | | | | | | |
| --- | --- | --- | --- | --- | --- | --- | --- |
| **Block** | **SNPs** | **Haplotype** | **Frequency** | **BETA** | **STAT** | ***p*-value** | **Adjusted *p*-value** |
| 1 | rs2167270, rs2278815 | GA | 0.158 | -0.0398 | 0.0128 | 0.91 | 1 |
|  |  | AG | 0.386 | 0.19 | 0.561 | 0.454 | 0.9988 |
|  |  | GG | 0.456 | -0.165 | 0.434 | 0.51 | 0.9998 |
| 2 | rs4731427, rs12706832, rs11763517, rs7795794, rs7796202, rs11760956, rs17151922 | AAAGGGA | 0.266 | -0.298 | 1.12 | 0.29 | 0.9778 |
|  |  | AAAGAGC | 0.139 | -0.653 | 3.26 | 0.0715 | 0.5881 |
|  |  | GAAAGGC | 0.122 | 0.579 | 2.22 | 0.137 | 0.8058 |
|  |  | AGAGGGC | 0.187 | 0.0038 | 0.00014 | 0.991 | 1 |
|  |  | AAGGGAC | 0.177 | 0.544 | 2.74 | 0.0981 | 0.7009 |
|  |  | AAAGGGC | 0.0186 | -0.373 | 0.16 | 0.69 | 1 |
|  |  | GAAGGGC | 0.0469 | 0.0234 | 0.00151 | 0.969 | 1 |
|  |  | AAAAGGC | 0.014 | 0.135 | 0.0156 | 0.901 | 1 |
|  |  | AGGGGGC | 0.0273 | 0.296 | 0.143 | 0.706 | 1 |
| 3 | rs28959474, rs2060715 | GG | 0.205 | -0.152 | 0.243 | 0.622 | 1 |
|  |  | AA | 0.117 | 0.344 | 0.798 | 0.372 | 0.9938 |
|  |  | GA | 0.678 | -0.0501 | 0.0363 | 0.849 | 1 |
| *n*=930  Abbreviations: LEP- leptin gene, SNP- single nucleotide polymorphism, STAT- test statistic, BETA- regression coefficient | | | | | | | |

| ***LEPR* Haplotype Association Analysis of Current Alcohol Use Disorder in European American Group** | | | | | | | |
| --- | --- | --- | --- | --- | --- | --- | --- |
| **Block** | **SNPs** | **Haplotype** | **Frequency** | **Odds Ratio** | **STAT** | ***p*-value** | **Adjusted *p*-value** |
| 1 | rs3806318, rs12145690, rs9436297 | AAG | 0.135 | 0.954 | 0.128 | 0.721 | 1 |
|  |  | GCA | 0.263 | 1.13 | 1.5 | 0.221 | 0.9948 |
|  |  | ACA | 0.204 | 1.04 | 0.156 | 0.693 | 1 |
|  |  | AAA | 0.395 | 0.88 | 1.9 | 0.168 | 0.978 |
| 2 | rs7534511, rs9436301, rs12065099, rs11208648, rs1887285, rs970467 | GGAAAA | 0.124 | 1.38 | 5.35 | 0.0207 | 0.3887 |
|  |  | GGAAGG | 0.0997 | 0.826 | 1.59 | 0.207 | 0.992 |
|  |  | GAGGAG | 0.0501 | 1 | 1.37E-06 | 0.999 | 1 |
|  |  | GGAAAG | 0.0355 | 1.59 | 3.03 | 0.0818 | 0.8392 |
|  |  | AAAAAG | 0.315 | 1.09 | 0.735 | 0.391 | 1 |
|  |  | GAAAAG | 0.372 | 0.806 | 5.35 | 0.0207 | 0.3885 |
| 3 | rs9436748, rs6657868, rs6669354 | CGC | 0.128 | 1.42 | 6.55 | 0.0105 | 0.2248 |
|  |  | CAA | 0.381 | 1.15 | 2.38 | 0.123 | 0.9336 |
|  |  | AGA | 0.399 | 0.81 | 5.29 | 0.0214 | 0.4001 |
|  |  | CGA | 0.0892 | 0.737 | 3.5 | 0.0615 | 0.7417 |
| 4 | rs6588147, rs12409877, rs2025803, rs7513047, rs11808888, rs17127673, rs11579567, rs1327121, rs6694528, rs11208654 | AGGAGACAGACG | 0.444 | 0.868 | 2.43 | 0.119 | 0.928 |
|  |  | AGAGAGCAAACG | 0.149 | 1.06 | 0.187 | 0.666 | 1 |
|  |  | GAAGGACGGGAA | 0.262 | 1.28 | 5.85 | 0.0155 | 0.3085 |
|  |  | GAAGGACGGGAG | 0.0624 | 0.817 | 1.18 | 0.278 | 0.999 |
|  |  | AAAGGAAAGAAG | 0.0609 | 0.843 | 0.791 | 0.374 | 1 |
| 5 | rs11208659, rs1782755, rs1171278, rs6697315, rs10749753, rs1177681, rs12140910, rs10158279, rs3790427 | AAAAAAAAA | 0.155 | 0.97 | 0.0625 | 0.803 | 1 |
|  |  | ACGAGAAAA | 0.326 | 0.872 | 1.81 | 0.179 | 0.9838 |
|  |  | ACGGAGACG | 0.249 | 1.25 | 4.59 | 0.0322 | 0.5219 |
|  |  | AAAAAAACA | 0.0241 | 0.836 | 0.354 | 0.552 | 1 |
|  |  | ACGAGAACA | 0.125 | 0.925 | 0.348 | 0.555 | 1 |
|  |  | GCGGAAGCA | 0.0583 | 0.804 | 1.23 | 0.268 | 0.9984 |
|  |  | GCGGAAACA | 0.0237 | 0.793 | 0.634 | 0.426 | 1 |
|  |  | AAAGAGACG | 0.0108 | 1.57 | 0.862 | 0.353 | 1 |
| 6 | rs3790426, rs11208675, rs10493380, rs12059300, rs6673591, rs1938489, rs12042877 | CAAGAAG | 0.318 | 0.983 | 0.0316 | 0.859 | 1 |
|  |  | CACGAAG | 0.188 | 0.897 | 0.882 | 0.348 | 1 |
|  |  | CCAGGAA | 0.282 | 1.23 | 4.08 | 0.0435 | 0.6249 |
|  |  | AAAAGGG | 0.211 | 0.875 | 1.52 | 0.218 | 0.9942 |
| 7 | rs2154380, rs1137101, rs4655537, rs12405556 | GGGA | 0.263 | 1.2 | 3.09 | 0.0786 | 0.824 |
|  |  | AAAC | 0.341 | 0.978 | 0.0559 | 0.813 | 1 |
|  |  | GGGC | 0.195 | 1.03 | 0.0503 | 0.823 | 1 |
|  |  | AAGC | 0.201 | 0.809 | 3.51 | 0.0612 | 0.7397 |
| 8 | rs4606347, rs8179183, rs6678033, rs17415296, rs1805096, rs1892534 | ACAAAA | 0.179 | 0.811 | 3.05 | 0.0807 | 0.8354 |
|  |  | GGACAA | 0.201 | 1.07 | 0.399 | 0.528 | 1 |
|  |  | GGGCGG | 0.604 | 1.12 | 1.53 | 0.217 | 0.994 |
| *n*=622 cases, 518 controls and 37 missing  Abbreviations: LEPR- leptin receptor gene, SNP- single nucleotide polymorphism, STAT- test statistic | | | | | | | |

| ***LEPR* Haplotype Association Analysis of Current Alcohol Use Disorder in African American Group** | | | | | | | |
| --- | --- | --- | --- | --- | --- | --- | --- |
| **Block** | **SNPs** | **Haplotype** | **Frequency** | **Odds Ratio** | **STAT** | ***p*-value** | **Adjusted *p*-value** |
| 1 | rs1214569, rs9436297 | AG | 0.139 | 1.03 | 0.0446 | 0.833 | 1 |
|  |  | CA | 0.42 | 0.972 | 0.0782 | 0.78 | 1 |
|  |  | AA | 0.441 | 1.01 | 0.0189 | 0.891 | 1 |
| 2 | rs12077210, rs3790433, rs7534511, rs9436301, rs12065099, rs11208648, rs1887285, rs970467 | GGAAAAAG | 0.194 | 0.926 | 0.346 | 0.557 | 1 |
|  |  | GAGGAAAA | 0.221 | 1.22 | 2.57 | 0.109 | 0.9678 |
|  |  | GAGGAAAG | 0.0486 | 1.14 | 0.296 | 0.586 | 1 |
|  |  | GAGGAAGG | 0.122 | 1.01 | 5.39E-03 | 0.941 | 1 |
|  |  | GGGAAAAG | 0.0716 | 0.907 | 0.221 | 0.638 | 1 |
|  |  | AAGAGGAG | 0.285 | 0.92 | 0.531 | 0.466 | 1 |
|  |  | GAGAGGAG | 0.0299 | 1.1 | 0.0947 | 0.758 | 1 |
|  |  | GAGAGGAA | 0.0108 | 1.24 | 0.163 | 0.686 | 1 |
| 3 | rs9436746, rs9436748, rs6657868, rs6669354 | CCGC | 0.152 | 1.01 | 0.00767 | 0.93 | 1 |
|  |  | ACAA | 0.472 | 0.995 | 0.00253 | 0.96 | 1 |
|  |  | CAGA | 0.188 | 1.14 | 0.934 | 0.334 | 1 |
|  |  | CCGA | 0.0365 | 0.728 | 1.47 | 0.225 | 0.9992 |
|  |  | ACGA | 0.135 | 1.07 | 0.212 | 0.646 | 1 |
| 4 | rs2025803, rs7513047, rs11808888, rs17127673, rs11579567, rs1327121, rs6694528, rs11208654, rs1327115 | AGGACGGGA | 0.201 | 1.04 | 0.0884 | 0.766 | 1 |
|  |  | AGAGCAAAC | 0.329 | 0.967 | 0.0943 | 0.759 | 1 |
|  |  | AGGAAAGAA | 0.157 | 0.95 | 0.125 | 0.724 | 1 |
|  |  | GAGACAGAC | 0.0917 | 0.944 | 0.0953 | 0.758 | 1 |
|  |  | AGAACAGAC | 0.0973 | 1.24 | 1.52 | 0.218 | 0.9988 |
|  |  | AGAACAAAC | 0.0733 | 0.891 | 0.343 | 0.558 | 1 |
|  |  | AGGACAGAA | 0.0309 | 1.49 | 1.76 | 0.184 | 0.9978 |
| 5 | rs10889557, rs2767485 | GG | 0.292 | 0.963 | 0.119 | 0.73 | 1 |
|  |  | AA | 0.412 | 1.07 | 0.406 | 0.524 | 1 |
|  |  | GA | 0.296 | 0.971 | 0.072 | 0.789 | 1 |
| 6 | rs1782755, rs1171278 | AA | 0.445 | 0.914 | 0.781 | 0.377 | 1 |
|  |  | CG | 0.553 | 1.11 | 1.11 | 0.293 | 1 |
| 7 | rs9660088, rs10158279, rs3790427, rs3790426, rs11208675, rs10493380, rs12059300, rs6673591 | ACAAAAAG | 0.109 | 0.92 | 0.257 | 0.612 | 1 |
|  |  | GAACAAGA | 0.428 | 0.967 | 0.107 | 0.744 | 1 |
|  |  | GCGCCAGG | 0.168 | 1.08 | 0.368 | 0.544 | 1 |
|  |  | GCAAAAAG | 0.0876 | 1.01 | 0.00605 | 0.938 | 1 |
|  |  | GAACACGA | 0.0552 | 0.977 | 0.0117 | 0.914 | 1 |
|  |  | GCACCAGG | 0.0727 | 0.964 | 0.0375 | 0.846 | 1 |
|  |  | GCAAAAGG | 0.0172 | 0.827 | 0.27 | 0.603 | 1 |
|  |  | GCAACAGG | 0.0155 | 1.06 | 0.023 | 0.879 | 1 |
|  |  | GCACCCGG | 0.0121 | 1.26 | 0.22 | 0.639 | 1 |
| 8 | rs1938489, rs12042877 | AA | 0.274 | 1.12 | 1.08 | 0.299 | 1 |
|  |  | GG | 0.226 | 0.903 | 0.707 | 0.4 | 1 |
|  |  | AG | 0.5 | 0.977 | 0.0531 | 0.818 | 1 |
| 9 | rs2154380, rs1137101, rs4655537 | AAA | 0.402 | 0.991 | 0.0081 | 0.928 | 1 |
|  |  | AAG | 0.0546 | 0.902 | 0.213 | 0.645 | 1 |
|  |  | GGG | 0.541 | 1.02 | 0.037 | 0.847 | 1 |
| 10 | rs12405556, rs4606347, rs8179183 | CAC | 0.186 | 0.881 | 0.905 | 0.342 | 1 |
|  |  | AGG | 0.163 | 1.17 | 1.31 | 0.253 | 1 |
|  |  | CGG | 0.65 | 0.988 | 0.0125 | 0.911 | 1 |
| 11 | rs4567312, rs17415296, rs1805096, rs1892534 | GAAA | 0.0631 | 0.839 | 0.733 | 0.392 | 1 |
|  |  | ACAA | 0.134 | 1.19 | 1.34 | 0.246 | 0.9998 |
|  |  | GCAA | 0.258 | 1.09 | 0.513 | 0.474 | 1 |
|  |  | GCGG | 0.543 | 0.916 | 0.688 | 0.407 | 1 |
| *n*=562 cases, 344 controls and 65 missing  Abbreviations: LEPR- leptin receptor gene, SNP- single nucleotide polymorphism, STAT- test statistic | | | | | | | |

| ***LEPR* Haplotype Association Analysis for Heavy Drinking Days in European American Group** | | | | | | | |
| --- | --- | --- | --- | --- | --- | --- | --- |
| **Block** | **SNPs** | **Haplotype** | **Frequency** | **BETA** | **STAT** | ***p*-value** | **Adjusted *p*-value** |
| 1 | rs3806318, rs12145690, rs9436297 | AAG | 0.135 | -0.711 | 0.117 | 0.733 | 1 |
|  |  | GCA | 0.263 | 1.34 | 0.726 | 0.394 | 1 |
|  |  | ACA | 0.204 | 1.01 | 0.329 | 0.566 | 1 |
|  |  | AAA | 0.395 | -1.32 | 0.825 | 0.364 | 1 |
| 2 | rs7534511, rs9436301, rs12065099, rs11208648, rs1887285, rs970467 | GGAAAA | 0.124 | 2.22 | 1.08 | 0.299 | 0.9992 |
|  |  | GGAAGG | 0.0997 | -3.35 | 1.96 | 0.162 | 0.9718 |
|  |  | GAGGAG | 0.0501 | 1.37 | 0.168 | 0.682 | 1 |
|  |  | GGAAAG | 0.0355 | 5.23 | 1.68 | 0.195 | 0.9874 |
|  |  | AAAAAG | 0.315 | 1.81 | 1.39 | 0.239 | 0.9956 |
|  |  | GAAAAG | 0.372 | -2.35 | 2.58 | 0.109 | 0.9106 |
| 3 | rs9436748, rs6657868, rs6669354 | CGC | 0.128 | 3.27 | 2.43 | 0.12 | 0.926 |
|  |  | CAA | 0.381 | 3.06 | 4.42 | 0.0358 | 0.5519 |
|  |  | AGA | 0.399 | -2.64 | 3.36 | 0.0673 | 0.7674 |
|  |  | CGA | 0.0892 | -6.4 | 6.21 | 0.0129 | 0.2687 |
| 4 | rs6588147, rs12409877, rs2025803, rs7513047, rs11808888, rs17127673, rs11579567, rs1327121, rs6694528, rs11208654, rs1327115, rs10889557, | AGGAGACAGACG | 0.444 | -1.7 | 1.4 | 0.237 | 0.9954 |
|  |  | AGAGAGCAAACG | 0.149 | -1.05 | 0.268 | 0.605 | 1 |
|  |  | GAAGGACGGGAA | 0.262 | 3.74 | 5.46 | 0.0197 | 0.3771 |
|  |  | GAAGGACGGGAG | 0.0624 | -2.13 | 0.511 | 0.475 | 1 |
|  |  | AAAGGAAAGAAG | 0.0609 | -3.45 | 1.24 | 0.266 | 0.9988 |
| 5 | rs11208659, rs1782755, rs1171278, rs6697315, rs10749753, rs1177681, rs12140910, rs10158279, rs3790427 | AAAAAAAAA | 0.155 | -2.83 | 2.19 | 0.139 | 0.9534 |
|  |  | ACGAGAAAA | 0.326 | -1.98 | 1.56 | 0.212 | 0.9908 |
|  |  | ACGGAGACG | 0.249 | 2.89 | 3.17 | 0.0754 | 0.8058 |
|  |  | AAAAAAACA | 0.0241 | 1.85 | 0.151 | 0.698 | 1 |
|  |  | ACGAGAACA | 0.125 | 1.22 | 0.338 | 0.561 | 1 |
|  |  | GCGGAAGCA | 0.0583 | -2.04 | 0.404 | 0.525 | 1 |
|  |  | GCGGAAACA | 0.0237 | -1.04 | 0.0526 | 0.819 | 1 |
|  |  | AAAGAGACG | 0.0108 | 9.21 | 1.34 | 0.247 | 0.9972 |
| 6 | rs3790426, rs11208675, rs10493380, rs12059300, rs6673591, rs1938489, rs12042877 | CAAGAAG | 0.318 | -0.871 | 0.324 | 0.57 | 1 |
|  |  | CACGAAG | 0.188 | -4.08 | 5.02 | 0.0253 | 0.4487 |
|  |  | CCAGGAA | 0.282 | 3.4 | 4.57 | 0.0327 | 0.5247 |
|  |  | AAAAGGG | 0.211 | 0.576 | 0.108 | 0.743 | 1 |
| 7 | rs2154380, rs1137101, rs4655537, rs12405556 | GGGA | 0.263 | 2.51 | 2.41 | 0.121 | 0.9276 |
|  |  | AAAC | 0.341 | 0.566 | 0.144 | 0.704 | 1 |
|  |  | GGGC | 0.195 | 0.488 | 0.0726 | 0.788 | 1 |
|  |  | AAGC | 0.201 | -4.31 | 5.91 | 0.0153 | 0.3089 |
| 9 | rs4606347, rs8179183, rs6678033, rs17415296, rs1805096, rs1892534 | ACAAAA | 0.179 | -4.89 | 6.69 | 0.00985 | 0.2166 |
|  |  | GGACAA | 0.201 | 0.88 | 0.242 | 0.623 | 1 |
|  |  | GGGCGG | 0.604 | 3 | 4.13 | 0.0424 | 0.6095 |
| *n*=1037  Abbreviations: LEPR- leptin receptor gene, SNP- single nucleotide polymorphism, STAT- test statistic, BETA- regression coefficient | | | | | | | |

| ***LEPR* Haplotype Association Analysis of Heavy Drinking Days in African American Group** | | | | | | | |
| --- | --- | --- | --- | --- | --- | --- | --- |
| **Block** | **SNPs** | **Haplotype** | **Frequency** | **BETA** | **STAT** | ***p*-value** | **Adjusted *p*-value** |
| 1 | rs1214569, rs9436297 | AG | 0.139 | -0.864 | 0.125 | 0.724 | 1 |
|  |  | CA | 0.42 | -0.679 | 0.17 | 0.68 | 1 |
|  |  | AA | 0.441 | 1.1 | 0.435 | 0.51 | 1 |
| 2 | rs12077210, rs3790433, rs7534511, rs9436301, rs12065099, rs11208648, rs1887285, rs970467 | GGAAAAAG | 0.194 | -3.05 | 1.97 | 0.161 | 0.9958 |
|  |  | GAGGAAAA | 0.221 | 1.55 | 0.591 | 0.442 | 1 |
|  |  | GAGGAAAG | 0.0486 | 2.95 | 0.549 | 0.459 | 1 |
|  |  | GAGGAAGG | 0.122 | -0.503 | 0.0384 | 0.845 | 1 |
|  |  | GGGAAAAG | 0.0716 | -3.56 | 1.1 | 0.294 | 1 |
|  |  | AAGAGGAG | 0.285 | 0.876 | 0.204 | 0.652 | 1 |
|  |  | GAGAGGAG | 0.0299 | 6.97 | 2.17 | 0.141 | 0.991 |
|  |  | GAGAGGAA | 0.0108 | -7.66 | 0.784 | 0.376 | 1 |
| 3 | rs9436746, rs9436748, rs6657868, rs6669354 | CCGC | 0.152 | -1.14 | 0.225 | 0.636 | 1 |
|  |  | ACAA | 0.472 | 0.824 | 0.244 | 0.621 | 1 |
|  |  | CAGA | 0.188 | -0.511 | 0.0568 | 0.812 | 1 |
|  |  | CCGA | 0.0365 | 0.378 | 0.00725 | 0.932 | 1 |
|  |  | ACGA | 0.135 | 1.12 | 0.208 | 0.649 | 1 |
| 4 | rs2025803, rs7513047, rs11808888, rs17127673, rs11579567, rs1327121, rs6694528, rs11208654, rs1327115 | AGGACGGGA | 0.201 | -1.31 | 0.379 | 0.538 | 1 |
|  |  | AGAGCAAAC | 0.329 | 0.384 | 0.0457 | 0.831 | 1 |
|  |  | AGGAAAGAA | 0.157 | 2.18 | 0.856 | 0.355 | 1 |
|  |  | GAGACAGAC | 0.0917 | -2.38 | 0.614 | 0.433 | 1 |
|  |  | AGAACAGAC | 0.0973 | 1.85 | 0.43 | 0.512 | 1 |
|  |  | AGAACAAAC | 0.0733 | 1.7 | 0.258 | 0.611 | 1 |
|  |  | AGGACAGAA | 0.0309 | -0.45 | 0.00821 | 0.928 | 1 |
| 5 | rs10889557, rs2767485 | GG | 0.292 | -0.641 | 0.126 | 0.722 | 1 |
|  |  | AA | 0.412 | 0.0521 | 0.000925 | 0.976 | 1 |
|  |  | GA | 0.296 | 0.662 | 0.131 | 0.718 | 1 |
| 6 | rs1782755, rs1171278 | AA | 0.445 | -1.13 | 0.447 | 0.504 | 1 |
|  |  | CG | 0.553 | 1.43 | 0.723 | 0.396 | 1 |
| 7 | rs9660088, rs10158279, rs3790427, rs3790426, rs11208675, rs10493380, rs12059300, rs6673591 | ACAAAAAG | 0.109 | -0.403 | 0.0213 | 0.884 | 1 |
|  |  | GAACAAGA | 0.428 | -0.529 | 0.0974 | 0.755 | 1 |
|  |  | GCGCCAGG | 0.168 | -0.648 | 0.0958 | 0.757 | 1 |
|  |  | GCAAAAAG | 0.0876 | -2.46 | 0.702 | 0.402 | 1 |
|  |  | GAACACGA | 0.0552 | 3.48 | 0.906 | 0.342 | 1 |
|  |  | GCACCAGG | 0.0727 | 1.28 | 0.176 | 0.675 | 1 |
|  |  | GCAAAAGG | 0.0172 | 1.16 | 0.0332 | 0.855 | 1 |
|  |  | GCAACAGG | 0.0155 | 3.7 | 0.291 | 0.59 | 1 |
|  |  | GCACCCGG | 0.0121 | 2.39 | 0.095 | 0.758 | 1 |
| 8 | rs1938489, rs12042877 | AA | 0.274 | 1.42 | 0.584 | 0.445 | 1 |
|  |  | GG | 0.226 | -2.29 | 1.28 | 0.258 | 1 |
|  |  | AG | 0.5 | 0.402 | 0.0601 | 0.806 | 1 |
| 9 | rs2154380, rs1137101, rs4655537 | AAA | 0.402 | 0.632 | 0.142 | 0.707 | 1 |
|  |  | AAG | 0.0546 | 2.36 | 0.381 | 0.537 | 1 |
|  |  | GGG | 0.541 | -1.07 | 0.413 | 0.521 | 1 |
| 10 | rs12405556, rs4606347, rs8179183 | CAC | 0.186 | -0.574 | 0.0671 | 0.796 | 1 |
|  |  | AGG | 0.163 | 0.173 | 0.00603 | 0.938 | 1 |
|  |  | CGG | 0.65 | 0.235 | 0.0183 | 0.893 | 1 |
| 11 | rs4567312, rs17415296, rs1805096, rs1892534 | GAAA | 0.0631 | -1.22 | 0.122 | 0.727 | 1 |
|  |  | ACAA | 0.134 | 0.411 | 0.0289 | 0.865 | 1 |
|  |  | GCAA | 0.258 | 0.979 | 0.25 | 0.617 | 1 |
|  |  | GCGG | 0.543 | -0.525 | 0.0897 | 0.765 | 1 |
| *n*=854  Abbreviations: LEPR- leptin receptor gene, SNP- single nucleotide polymorphism, STAT- test statistic, BETA- regression coefficient | | | | | | | |

| ***LEPR* Haplotype Association Analysis of Average Drinks/Drinking Day in European American Group** | | | | | | | |
| --- | --- | --- | --- | --- | --- | --- | --- |
| **Block** | **SNPs** | **Haplotype** | **Frequency** | **BETA** | **STAT** | ***p*-value** | **Adjusted *p*-value** |
| 1 | rs3806318, rs12145690, rs9436297 | AAG | 0.135 | -0.433 | 0.727 | 0.394 | 0.9996 |
|  |  | GCA | 0.263 | 0.0445 | 0.0135 | 0.908 | 1 |
|  |  | ACA | 0.204 | 0.358 | 0.69 | 0.406 | 0.9998 |
|  |  | AAA | 0.395 | -0.0317 | 0.00799 | 0.929 | 1 |
| 2 | rs7534511, rs9436301, rs12065099, rs11208648, rs1887285, rs970467 | GGAAAA | 0.124 | -0.11 | 0.0447 | 0.833 | 1 |
|  |  | GGAAGG | 0.0997 | -0.441 | 0.568 | 0.451 | 1 |
|  |  | GAGGAG | 0.0501 | -0.71 | 0.766 | 0.382 | 0.9996 |
|  |  | GGAAAG | 0.0355 | 1.74 | 3.16 | 0.0757 | 0.8076 |
|  |  | AAAAAG | 0.315 | 0.158 | 0.179 | 0.672 | 1 |
|  |  | GAAAAG | 0.372 | 0.0201 | 0.00316 | 0.955 | 1 |
| 3 | rs9436748, rs6657868, rs6669354 | CGC | 0.128 | 0.143 | 0.0778 | 0.78 | 1 |
|  |  | CAA | 0.381 | 0.327 | 0.852 | 0.356 | 0.9996 |
|  |  | AGA | 0.399 | -0.0778 | 0.0489 | 0.825 | 1 |
|  |  | CGA | 0.0892 | -1.07 | 2.91 | 0.0885 | 0.8492 |
| 4 | rs6588147, rs12409877, rs2025803, rs7513047, rs11808888, rs17127673, rs11579567, rs1327121, rs6694528, rs11208654, rs1327115, rs10889557, | AGGAGACAGACG | 0.444 | -0.0435 | 0.0156 | 0.901 | 1 |
|  |  | AGAGAGCAAACG | 0.149 | -0.203 | 0.168 | 0.682 | 1 |
|  |  | GAAGGACGGGAA | 0.262 | 0.626 | 2.57 | 0.109 | 0.9056 |
|  |  | GAAGGACGGGAG | 0.0624 | -0.431 | 0.354 | 0.552 | 1 |
|  |  | AAAGGAAAGAAG | 0.0609 | -2.09 | 7.73 | 0.0055 | 0.1352 |
| 5 | rs11208659, rs1782755, rs1171278, rs6697315, rs10749753, rs1177681, rs12140910, rs10158279, rs3790427 | AAAAAAAAA | 0.155 | -0.311 | 0.442 | 0.506 | 1 |
|  |  | ACGAGAAAA | 0.326 | -0.0318 | 0.00676 | 0.934 | 1 |
|  |  | ACGGAGACG | 0.249 | 0.639 | 2.6 | 0.107 | 0.9022 |
|  |  | AAAAAAACA | 0.0241 | 0.229 | 0.0385 | 0.844 | 1 |
|  |  | ACGAGAACA | 0.125 | -0.0815 | 0.0253 | 0.874 | 1 |
|  |  | GCGGAAGCA | 0.0583 | -1.71 | 4.85 | 0.0278 | 0.4733 |
|  |  | GCGGAAACA | 0.0237 | 0.589 | 0.288 | 0.592 | 1 |
|  |  | AAAGAGACG | 0.0108 | 1.67 | 0.746 | 0.388 | 0.9996 |
| 6 | rs3790426, rs11208675, rs10493380, rs12059300, rs6673591, rs1938489, rs12042877 | CAAGAAG | 0.318 | 0.157 | 0.175 | 0.676 | 1 |
|  |  | CACGAAG | 0.188 | -0.709 | 2.54 | 0.111 | 0.9096 |
|  |  | CCAGGAA | 0.282 | 0.784 | 4.1 | 0.0431 | 0.6171 |
|  |  | AAAAGGG | 0.211 | -0.565 | 1.75 | 0.186 | 0.9816 |
| 7 | rs2154380, rs1137101, rs4655537, rs12405556 | GGGA | 0.263 | 0.545 | 1.91 | 0.167 | 0.9722 |
|  |  | AAAC | 0.341 | 0.121 | 0.11 | 0.74 | 1 |
|  |  | GGGC | 0.195 | -0.247 | 0.314 | 0.575 | 1 |
|  |  | AAGC | 0.201 | -0.591 | 1.86 | 0.173 | 0.9754 |
| 9 | rs4606347, rs8179183, rs6678033, rs17415296, rs1805096, rs1892534 | ACAAAA | 0.179 | -0.879 | 3.64 | 0.0566 | 0.7153 |
|  |  | GGACAA | 0.201 | 0.297 | 0.465 | 0.495 | 1 |
|  |  | GGGCGG | 0.604 | 0.439 | 1.49 | 0.223 | 0.9916 |
| *n*=1032  Abbreviations: LEPR- leptin receptor gene, SNP- single nucleotide polymorphism, STAT- test statistic, BETA- regression coefficient | | | | | | | |

| ***LEPR* Haplotype Association Analysis of Average Drinks/Drinking Day in African American Group** | | | | | | | |
| --- | --- | --- | --- | --- | --- | --- | --- |
| **Block** | **SNPs** | **Haplotype** | **Frequency** | **BETA** | **STAT** | ***p*-value** | **Adjusted *p*-value** |
| 1 | rs1214569, rs9436297 | AG | 0.139 | 0.212 | 0.134 | 0.715 | 1 |
|  |  | CA | 0.42 | -0.221 | 0.32 | 0.571 | 1 |
|  |  | AA | 0.441 | 0.128 | 0.105 | 0.746 | 1 |
| 2 | rs12077210, rs3790433, rs7534511, rs9436301, rs12065099, rs11208648, rs1887285, rs970467 | GGAAAAAG | 0.194 | -0.68 | 1.75 | 0.186 | 0.9976 |
|  |  | GAGGAAAA | 0.221 | 0.222 | 0.217 | 0.642 | 1 |
|  |  | GAGGAAAG | 0.0486 | 0.128 | 0.0188 | 0.891 | 1 |
|  |  | GAGGAAGG | 0.122 | -0.59 | 0.951 | 0.33 | 1 |
|  |  | GGGAAAAG | 0.0716 | 0.608 | 0.576 | 0.448 | 1 |
|  |  | AAGAGGAG | 0.285 | 0.593 | 1.67 | 0.196 | 0.9984 |
|  |  | GAGAGGAG | 0.0299 | 0.481 | 0.183 | 0.669 | 1 |
|  |  | GAGAGGAA | 0.0108 | -2.24 | 1.21 | 0.272 | 1 |
| 3 | rs9436746, rs9436748, rs6657868, rs6669354 | CCGC | 0.152 | -0.0457 | 0.00653 | 0.936 | 1 |
|  |  | ACAA | 0.472 | 0.327 | 0.689 | 0.407 | 1 |
|  |  | CAGA | 0.188 | 0.0327 | 0.00418 | 0.948 | 1 |
|  |  | CCGA | 0.0365 | -0.804 | 0.593 | 0.441 | 1 |
|  |  | ACGA | 0.135 | -0.232 | 0.16 | 0.69 | 1 |
| 4 | rs2025803, rs7513047, rs11808888, rs17127673, rs11579567, rs1327121, rs6694528, rs11208654, rs1327115 | AGGACGGGA | 0.201 | -0.452 | 0.805 | 0.37 | 1 |
|  |  | AGAGCAAAC | 0.329 | -0.0949 | 0.05 | 0.823 | 1 |
|  |  | AGGAAAGAA | 0.157 | 0.0724 | 0.0166 | 0.897 | 1 |
|  |  | GAGACAGAC | 0.0917 | 0.825 | 1.33 | 0.249 | 1 |
|  |  | AGAACAGAC | 0.0973 | -0.392 | 0.348 | 0.555 | 1 |
|  |  | AGAACAAAC | 0.0733 | 1.63 | 4.2 | 0.0408 | 0.755 |
|  |  | AGGACAGAA | 0.0309 | -0.35 | 0.0897 | 0.765 | 1 |
| 5 | rs10889557, rs2767485 | GG | 0.292 | 0.215 | 0.254 | 0.614 | 1 |
|  |  | AA | 0.412 | -0.492 | 1.47 | 0.225 | 0.9998 |
|  |  | GA | 0.296 | 0.35 | 0.654 | 0.419 | 1 |
| 6 | rs1782755, rs1171278 | AA | 0.445 | -0.0416 | 0.0109 | 0.917 | 1 |
|  |  | CG | 0.553 | 0.0976 | 0.0601 | 0.806 | 1 |
| 7 | rs9660088, rs10158279, rs3790427, rs3790426, rs11208675, rs10493380, rs12059300, rs6673591 | ACAAAAAG | 0.109 | -0.38 | 0.341 | 0.559 | 1 |
|  |  | GAACAAGA | 0.428 | 0.256 | 0.411 | 0.522 | 1 |
|  |  | GCGCCAGG | 0.168 | -0.772 | 2.47 | 0.116 | 0.9804 |
|  |  | GCAAAAAG | 0.0876 | 0.0846 | 0.015 | 0.902 | 1 |
|  |  | GAACACGA | 0.0552 | 1.31 | 2.34 | 0.126 | 0.986 |
|  |  | GCACCAGG | 0.0727 | 0.127 | 0.0303 | 0.862 | 1 |
|  |  | GCAAAAGG | 0.0172 | -0.924 | 0.38 | 0.538 | 1 |
|  |  | GCAACAGG | 0.0155 | -0.0934 | 0.00325 | 0.955 | 1 |
|  |  | GCACCCGG | 0.0121 | 2.27 | 1.49 | 0.223 | 0.9998 |
| 8 | rs1938489, rs12042877 | AA | 0.274 | -0.533 | 1.46 | 0.227 | 0.9998 |
|  |  | GG | 0.226 | -0.183 | 0.147 | 0.702 | 1 |
|  |  | AG | 0.5 | 0.534 | 1.9 | 0.169 | 0.9968 |
| 9 | rs2154380, rs1137101, rs4655537 | AAA | 0.402 | 0.248 | 0.392 | 0.532 | 1 |
|  |  | AAG | 0.0546 | 2.4 | 7.09 | 0.00791 | 0.2819 |
|  |  | GGG | 0.541 | -0.735 | 3.53 | 0.0608 | 0.8688 |
| 10 | rs12405556, rs4606347, rs8179183 | CAC | 0.186 | 0.511 | 0.954 | 0.329 | 1 |
|  |  | AGG | 0.163 | -0.55 | 1.09 | 0.297 | 1 |
|  |  | CGG | 0.65 | -0.0131 | 0.00101 | 0.975 | 1 |
| 11 | rs4567312, rs17415296, rs1805096, rs1892534 | GAAA | 0.0631 | 1.13 | 1.87 | 0.171 | 0.997 |
|  |  | ACAA | 0.134 | 0.279 | 0.235 | 0.628 | 1 |
|  |  | GCAA | 0.258 | -0.0831 | 0.0323 | 0.858 | 1 |
|  |  | GCGG | 0.543 | -0.331 | 0.637 | 0.425 | 1 |
| *n*=847  Abbreviations: LEPR- leptin receptor gene, SNP- single nucleotide polymorphism, STAT- test statistic, BETA- regression coefficient | | | | | | | |

| ***LEPR* Haplotype Association Analysis of Alcohol Use Disorders Identification Test Score in European American Group** | | | | | | | |
| --- | --- | --- | --- | --- | --- | --- | --- |
| **Block** | **SNPs** | **Haplotype** | **Frequency** | **BETA** | **STAT** | ***p*-value** | **Adjusted *p*-value** |
| 1 | rs3806318, rs12145690, rs9436297 | AAG | 0.135 | -0.661 | 0.656 | 0.418 | 0.9998 |
|  |  | GCA | 0.263 | 0.923 | 2.43 | 0.12 | 0.9252 |
|  |  | ACA | 0.204 | 0.521 | 0.646 | 0.422 | 1 |
|  |  | AAA | 0.395 | -0.988 | 3.17 | 0.0753 | 0.8112 |
| 2 | rs7534511, rs9436301, rs12065099, rs11208648, rs1887285, rs970467 | GGAAAA | 0.124 | 0.284 | 0.112 | 0.738 | 1 |
|  |  | GGAAGG | 0.0997 | -0.864 | 0.967 | 0.326 | 0.9998 |
|  |  | GAGGAG | 0.0501 | 1.24 | 1.01 | 0.315 | 0.9998 |
|  |  | GGAAAG | 0.0355 | 3.74 | 5.74 | 0.0169 | 0.3417 |
|  |  | AAAAAG | 0.315 | 0.583 | 0.939 | 0.333 | 0.9998 |
|  |  | GAAAAG | 0.372 | -0.958 | 2.99 | 0.0843 | 0.8412 |
| 3 | rs9436748, rs6657868, rs6669354 | CGC | 0.128 | 1.04 | 1.56 | 0.212 | 0.9902 |
|  |  | CAA | 0.381 | 1.17 | 4.24 | 0.0398 | 0.5953 |
|  |  | AGA | 0.399 | -1.11 | 4.07 | 0.0439 | 0.6327 |
|  |  | CGA | 0.0892 | -1.56 | 2.74E+00 | 0.0985 | 0.8808 |
| 4 | rs6588147, rs12409877, rs2025803, rs7513047, rs11808888, rs17127673, rs11579567, rs1327121, rs6694528, rs11208654, rs1327115, rs10889557, | AGGAGACAGACG | 0.444 | -0.796 | 2.11 | 0.147 | 0.9592 |
|  |  | AGAGAGCAAACG | 0.149 | -0.178 | 0.0516 | 0.82 | 1 |
|  |  | GAAGGACGGGAA | 0.262 | 1.19 | 3.4 | 0.0655 | 0.7674 |
|  |  | GAAGGACGGGAG | 0.0624 | 0.0833 | 0.0055 | 0.941 | 1 |
|  |  | AAAGGAAAGAAG | 0.0609 | -1.63 | 1.91 | 0.167 | 0.9722 |
| 5 | rs11208659, rs1782755, rs1171278, rs6697315, rs10749753, rs1177681, rs12140910, rs10158279, rs3790427 | AAAAAAAAA | 0.155 | 0.0033 | 2.08E-05 | 0.996 | 1 |
|  |  | ACGAGAAAA | 0.326 | -0.494 | 0.685 | 0.408 | 0.9998 |
|  |  | ACGGAGACG | 0.249 | 1.46 | 5.1 | 0.0242 | 0.4355 |
|  |  | AAAAAAACA | 0.0241 | -0.279 | 0.0233 | 0.879 | 1 |
|  |  | ACGAGAACA | 0.125 | -0.0888 | 0.0115 | 0.915 | 1 |
|  |  | GCGGAAGCA | 0.0583 | -1.16 | 0.928 | 0.336 | 0.9998 |
|  |  | GCGGAAACA | 0.0237 | -2.15 | 1.62 | 0.203 | 0.9884 |
|  |  | AAAGAGACG | 0.0108 | -2.82 | 0.589 | 0.443 | 1 |
| 6 | rs3790426, rs11208675, rs10493380, rs12059300, rs6673591, rs1938489, rs12042877 | CAAGAAG | 0.318 | -0.284 | 0.232 | 0.63 | 1 |
|  |  | CACGAAG | 0.188 | -0.423 | 0.381 | 0.537 | 1 |
|  |  | CCAGGAA | 0.282 | 1.02 | 2.58 | 0.109 | 0.9066 |
|  |  | AAAAGGG | 0.211 | -0.471 | 0.483 | 0.487 | 1 |
| 7 | rs2154380, rs1137101, rs4655537, rs12405556 | GGGA | 0.263 | 1.18 | 3.42 | 0.0647 | 0.765 |
|  |  | AAAC | 0.341 | -0.888 | 2.3 | 0.13 | 0.9386 |
|  |  | GGGC | 0.195 | 0.495 | 0.519 | 0.471 | 1 |
|  |  | AAGC | 0.201 | -0.569 | 0.769 | 0.381 | 0.9998 |
| 9 | rs4606347, rs8179183, rs6678033, rs17415296, rs1805096, rs1892534 | ACAAAA | 0.179 | -0.301 | 0.182 | 0.67 | 1 |
|  |  | GGACAA | 0.201 | 1.18 | 3.03 | 0.0823 | 0.835 |
|  |  | GGGCGG | 0.604 | -0.471 | 0.697 | 0.404 | 0.9998 |
| *n*=692  Abbreviations: LEPR- leptin receptor gene, SNP- single nucleotide polymorphism, STAT- test statistic, BETA- regression coefficient | | | | | | | |

| ***LEPR* Haplotype Association Analysis of Alcohol Use Disorders Identification Test Score in African American Group** | | | | | | | |
| --- | --- | --- | --- | --- | --- | --- | --- |
| **Block** | **SNPs** | **Haplotype** | **Frequency** | **BETA** | **STAT** | ***p*-value** | **Adjusted *p*-value** |
| 1 | rs1214569, rs9436297 | AG | 0.139 | -0.211 | 0.061 | 0.805 | 1 |
|  |  | CA | 0.42 | 0.188 | 0.106 | 0.744 | 1 |
|  |  | AA | 0.441 | -0.07 | 0.0142 | 0.905 | 1 |
| 2 | rs12077210, rs3790433, rs7534511, rs9436301, rs12065099, rs11208648, rs1887285, rs970467 | GGAAAAAG | 0.194 | -0.415 | 0.323 | 0.57 | 1 |
|  |  | GAGGAAAA | 0.221 | 0.719 | 1.05 | 0.306 | 1 |
|  |  | GAGGAAAG | 0.0486 | 0.21 | 0.0239 | 0.877 | 1 |
|  |  | GAGGAAGG | 0.122 | -0.0203 | 0.000541 | 0.981 | 1 |
|  |  | GGGAAAAG | 0.0716 | -0.679 | 0.33 | 0.566 | 1 |
|  |  | AAGAGGAG | 0.285 | 0.198 | 0.0864 | 0.769 | 1 |
|  |  | GAGAGGAG | 0.0299 | 0.882 | 0.333 | 0.564 | 1 |
|  |  | GAGAGGAA | 0.0108 | -2.11 | 0.56 | 0.454 | 1 |
| 3 | rs9436746, rs9436748, rs6657868, rs6669354 | CCGC | 0.152 | -0.878 | 1.12 | 0.29 | 1 |
|  |  | ACAA | 0.472 | 0.749 | 1.7 | 0.193 | 0.9982 |
|  |  | CAGA | 0.188 | 0.447 | 0.352 | 0.553 | 1 |
|  |  | CCGA | 0.0365 | -1.91 | 1.46 | 0.227 | 0.9998 |
|  |  | ACGA | 0.135 | 0.0881 | 0.011 | 0.916 | 1 |
| 4 | rs2025803, rs7513047, rs11808888, rs17127673, rs11579567, rs1327121, rs6694528, rs11208654, rs1327115 | AGGACGGGA | 0.201 | 0.0234 | 0.00103 | 0.974 | 1 |
|  |  | AGAGCAAAC | 0.329 | -0.309 | 0.242 | 0.623 | 1 |
|  |  | AGGAAAGAA | 0.157 | 0.74 | 0.806 | 0.37 | 1 |
|  |  | GAGACAGAC | 0.0917 | -0.0911 | 0.00753 | 0.931 | 1 |
|  |  | AGAACAGAC | 0.0973 | 1.63 | 2.76 | 0.0974 | 0.9604 |
|  |  | AGAACAAAC | 0.0733 | -1.43 | 1.5 | 0.222 | 0.9998 |
|  |  | AGGACAGAA | 0.0309 | 1.28 | 0.579 | 0.447 | 1 |
| 5 | rs10889557, rs2767485 | GG | 0.292 | -0.109 | 0.0313 | 0.86 | 1 |
|  |  | AA | 0.412 | -0.142 | 0.058 | 0.81 | 1 |
|  |  | GA | 0.296 | 0.305 | 0.23 | 0.632 | 1 |
| 6 | rs1782755, rs1171278 | AA | 0.445 | -0.523 | 0.773 | 0.38 | 1 |
|  |  | CG | 0.553 | 0.612 | 1.06 | 0.303 | 1 |
| 7 | rs9660088, rs10158279, rs3790427, rs3790426, rs11208675, rs10493380, rs12059300, rs6673591 | ACAAAAAG | 0.109 | -1.22 | 1.63 | 0.202 | 0.9996 |
|  |  | GAACAAGA | 0.428 | -0.275 | 0.223 | 0.637 | 1 |
|  |  | GCGCCAGG | 0.168 | 0.501 | 0.499 | 0.48 | 1 |
|  |  | GCAAAAAG | 0.0876 | 2.21 | 4.93 | 0.0268 | 0.6093 |
|  |  | GAACACGA | 0.0552 | 0.358 | 0.0763 | 0.783 | 1 |
|  |  | GCACCAGG | 0.0727 | -0.34 | 0.107 | 0.743 | 1 |
|  |  | GCAAAAGG | 0.0172 | -0.771 | 0.14 | 0.708 | 1 |
|  |  | GCAACAGG | 0.0155 | -1.6 | 0.512 | 0.475 | 1 |
|  |  | GCACCCGG | 0.0121 | -2.18 | 0.518 | 0.472 | 1 |
| 8 | rs1938489, rs12042877 | AA | 0.274 | -0.0423 | 0.00444 | 0.947 | 1 |
|  |  | GG | 0.226 | 0.262 | 0.15 | 0.699 | 1 |
|  |  | AG | 0.5 | -0.145 | 0.0676 | 0.795 | 1 |
| 9 | rs2154380, rs1137101, rs4655537 | AAA | 0.402 | 0.082 | 0.0198 | 0.888 | 1 |
|  |  | AAG | 0.0546 | -0.314 | 0.0578 | 0.81 | 1 |
|  |  | GGG | 0.541 | 0.0228 | 0.00159 | 0.968 | 1 |
| 10 | rs12405556, rs4606347, rs8179183 | CAC | 0.186 | -0.755 | 0.964 | 0.326 | 1 |
|  |  | AGG | 0.163 | 0.814 | 1.14 | 0.286 | 1 |
|  |  | CGG | 0.65 | -0.0937 | 0.0244 | 0.876 | 1 |
| 11 | rs4567312, rs17415296, rs1805096, rs1892534 | GAAA | 0.0631 | -0.0977 | 0.00689 | 0.934 | 1 |
|  |  | ACAA | 0.134 | 0.428 | 0.278 | 0.598 | 1 |
|  |  | GCAA | 0.258 | -0.752 | 1.2 | 0.274 | 1 |
|  |  | GCGG | 0.543 | 0.412 | 0.46 | 0.498 | 1 |
| *n*=631  Abbreviations: LEPR- leptin receptor gene, SNP- single nucleotide polymorphism, STAT- test statistic, BETA- regression coefficient | | | | | | | |

| ***LEPR* Haplotype Association Analysis of Cigarette Pack Years in European American Group** | | | | | | | |
| --- | --- | --- | --- | --- | --- | --- | --- |
| **Block** | **SNPs** | **Haplotype** | **Frequency** | **BETA** | **STAT** | ***p*-value** | **Adjusted *p*-value** |
| 1 | rs3806318, rs12145690, rs9436297 | AAG | 0.135 | -0.572 | 0.565 | 0.452 | 1 |
|  |  | GCA | 0.263 | -0.518 | 0.816 | 0.367 | 0.9998 |
|  |  | ACA | 0.204 | 1.01 | 2.47 | 0.116 | 0.9194 |
|  |  | AAA | 0.395 | -0.0171 | 0.00103 | 0.974 | 1 |
| 2 | rs7534511, rs9436301, rs12065099, rs11208648, rs1887285, rs970467 | GGAAAA | 0.124 | -0.198 | 0.0643 | 0.8 | 1 |
|  |  | GGAAGG | 0.0997 | -1.86 | 4.61 | 0.0319 | 0.5181 |
|  |  | GAGGAG | 0.0501 | -0.527 | 0.189 | 0.664 | 1 |
|  |  | GGAAAG | 0.0355 | 0.0303 | 0.000427 | 0.984 | 1 |
|  |  | AAAAAG | 0.315 | 1.28 | 5.26 | 0.022 | 0.4037 |
|  |  | GAAAAG | 0.372 | -0.209 | 0.156 | 0.693 | 1 |
| 3 | rs9436748, rs6657868, rs6669354 | CGC | 0.128 | -0.194 | 0.0637 | 0.801 | 1 |
|  |  | CAA | 0.381 | 1.22 | 5.28 | 0.0217 | 0.4015 |
|  |  | AGA | 0.399 | -0.514 | 0.97 | 0.325 | 0.9996 |
|  |  | CGA | 0.0892 | -1.83 | 3.85 | 0.0502 | 0.6749 |
| 4 | rs6588147, rs12409877, rs2025803, rs7513047, rs11808888, rs17127673, rs11579567, rs1327121, rs6694528, rs11208654, rs1327115, rs10889557 | AGGAGACAGACG | 0.444 | -0.843 | 2.62 | 0.106 | 0.9006 |
|  |  | AGAGAGCAAACG | 0.149 | -0.507 | 0.467 | 0.494 | 1 |
|  |  | GAAGGACGGGAA | 0.262 | 1.51 | 6.62 | 0.0102 | 0.2316 |
|  |  | GAAGGACGGGAG | 0.0624 | 0.297 | 0.076 | 0.783 | 1 |
|  |  | AAAGGAAAGAAG | 0.0609 | -1.07 | 0.899 | 0.343 | 0.9998 |
| 5 | rs11208659, rs1782755, rs1171278, rs6697315, rs10749753, rs1177681, rs12140910, rs10158279, rs3790427 | AAAAAAAAA | 0.155 | -0.969 | 1.93 | 0.165 | 0.9736 |
|  |  | ACGAGAAAA | 0.326 | -1.02 | 3.12 | 0.0775 | 0.8206 |
|  |  | ACGGAGACG | 0.249 | 1.42 | 5.64 | 0.0177 | 0.3495 |
|  |  | AAAAAAACA | 0.0241 | -0.109 | 0.00402 | 0.949 | 1 |
|  |  | ACGAGAACA | 0.125 | 0.237 | 0.0943 | 0.759 | 1 |
|  |  | GCGGAAGCA | 0.0583 | -1.13 | 0.951 | 0.33 | 0.9996 |
|  |  | GCGGAAACA | 0.0237 | 1.32 | 0.628 | 0.428 | 1 |
|  |  | AAAGAGACG | 0.0108 | 3.57 | 1.52 | 0.218 | 0.992 |
| 6 | rs3790426, rs11208675, rs10493380, rs12059300, rs6673591, rs1938489, rs12042877 | CAAGAAG | 0.318 | 0.00205 | 1.32E-05 | 0.997 | 1 |
|  |  | CACGAAG | 0.188 | -1.97 | 9.06 | 0.00267 | 0.0802 |
|  |  | CCAGGAA | 0.282 | 1.58 | 7.27 | 0.00711 | 0.1736 |
|  |  | AAAAGGG | 0.211 | -0.0156 | 0.000585 | 0.981 | 1 |
| 7 | rs2154380, rs1137101, rs4655537, rs12405556 | GGGA | 0.263 | 1.34 | 5.07 | 0.0245 | 0.4335 |
|  |  | AAAC | 0.341 | 0.135 | 0.0606 | 0.806 | 1 |
|  |  | GGGC | 0.195 | 0.564 | 0.728 | 0.394 | 1 |
|  |  | AAGC | 0.201 | -2.27 | 12.7 | 0.000391 | **0.0126** |
| 9 | rs4606347, rs8179183, rs6678033, rs17415296, rs1805096, rs1892534 | ACAAAA | 0.179 | -2.46 | 13.2 | 0.000293 | **0.0092** |
|  |  | GGACAA | 0.201 | 1.53 | 5.44 | 0.0199 | 0.3779 |
|  |  | GGGCGG | 0.604 | 0.638 | 1.39 | 0.238 | 0.9946 |
| *n*=1034  Abbreviations: LEPR- leptin receptor gene, SNP- single nucleotide polymorphism, STAT- test statistic, BETA- regression coefficient  Adjusted *p*-values in bold indicate statistically significant associations | | | | | | | |

| ***LEPR* Haplotype Association Analysis of Cigarette Pack Years in African American Group** | | | | | | | |
| --- | --- | --- | --- | --- | --- | --- | --- |
| **Block** | **SNPs** | **Haplotype** | **Frequency** | **BETA** | **STAT** | ***p*-value** | **Adjusted *p*-value** |
| 1 | rs1214569, rs9436297 | AG | 0.139 | 0.565 | 0.459 | 0.498 | 1 |
|  |  | CA | 0.42 | 0.306 | 0.298 | 0.585 | 1 |
|  |  | AA | 0.441 | -0.568 | 0.989 | 0.32 | 1 |
| 2 | rs12077210, rs3790433, rs7534511, rs9436301, rs12065099, rs11208648, rs1887285, rs970467 | GGAAAAAG | 0.194 | -1.38 | 3.48 | 0.0626 | 0.8714 |
|  |  | GAGGAAAA | 0.221 | 0.385 | 0.316 | 0.574 | 1 |
|  |  | GAGGAAAG | 0.0486 | -0.136 | 0.0101 | 0.92 | 1 |
|  |  | GAGGAAGG | 0.122 | 0.26 | 0.0861 | 0.769 | 1 |
|  |  | GGGAAAAG | 0.0716 | -1.65 | 2.01 | 0.156 | 0.9936 |
|  |  | AAGAGGAG | 0.285 | 0.49 | 0.556 | 0.456 | 1 |
|  |  | GAGAGGAG | 0.0299 | 2.02 | 1.65 | 0.2 | 0.9974 |
|  |  | GAGAGGAA | 0.0108 | 7.66 | 7.34 | 0.00688 | 0.3047 |
| 3 | rs9436746, rs9436748, rs6657868, rs6669354 | CCGC | 0.152 | 0.781 | 0.921 | 0.338 | 1 |
|  |  | ACAA | 0.472 | 0.275 | 0.233 | 0.629 | 1 |
|  |  | CAGA | 0.188 | -0.571 | 0.622 | 0.431 | 1 |
|  |  | CCGA | 0.0365 | -0.011 | 5.27E-05 | 0.994 | 1 |
|  |  | ACGA | 0.135 | -0.488 | 0.331 | 0.565 | 1 |
| 4 | rs2025803, rs7513047, rs11808888, rs17127673, rs11579567, rs1327121, rs6694528, rs11208654, rs1327115 | AGGACGGGA | 0.201 | -0.699 | 0.923 | 0.337 | 1 |
|  |  | AGAGCAAAC | 0.329 | 0.184 | 0.0903 | 0.764 | 1 |
|  |  | AGGAAAGAA | 0.157 | 1.45 | 3.27 | 0.071 | 0.9038 |
|  |  | GAGACAGAC | 0.0917 | -0.733 | 0.503 | 0.478 | 1 |
|  |  | AGAACAGAC | 0.0973 | 0.875 | 0.87 | 0.351 | 1 |
|  |  | AGAACAAAC | 0.0733 | -1.78 | 2.45 | 0.118 | 0.9774 |
|  |  | AGGACAGAA | 0.0309 | -0.333 | 0.038 | 0.845 | 1 |
| 5 | rs10889557, rs2767485 | GG | 0.292 | -0.199 | 0.104 | 0.748 | 1 |
|  |  | AA | 0.412 | -0.028 | 0.00235 | 0.961 | 1 |
|  |  | GA | 0.296 | 0.197 | 0.0994 | 0.753 | 1 |
| 6 | rs1782755, rs1171278 | AA | 0.445 | -0.588 | 1.04 | 0.309 | 1 |
|  |  | CG | 0.553 | 0.645 | 1.25 | 0.264 | 1 |
| 7 | rs9660088, rs10158279, rs3790427, rs3790426, rs11208675, rs10493380, rs12059300, rs6673591 | ACAAAAAG | 0.109 | 0.549 | 0.353 | 0.553 | 1 |
|  |  | GAACAAGA | 0.428 | -0.414 | 0.514 | 0.473 | 1 |
|  |  | GCGCCAGG | 0.168 | -0.146 | 0.0419 | 0.838 | 1 |
|  |  | GCAAAAAG | 0.0876 | -0.537 | 0.292 | 0.589 | 1 |
|  |  | GAACACGA | 0.0552 | -0.809 | 0.438 | 0.508 | 1 |
|  |  | GCACCAGG | 0.0727 | 2.08 | 3.91 | 0.0483 | 0.8066 |
|  |  | GCAAAAGG | 0.0172 | -1.06 | 0.233 | 0.63 | 1 |
|  |  | GCAACAGG | 0.0155 | -0.329 | 0.0215 | 0.884 | 1 |
|  |  | GCACCCGG | 0.0121 | 0.211 | 0.00615 | 0.938 | 1 |
| 8 | rs1938489, rs12042877 | AA | 0.274 | 0.924 | 2.13 | 0.145 | 0.9896 |
|  |  | GG | 0.226 | -0.402 | 0.351 | 0.554 | 1 |
|  |  | AG | 0.5 | -0.444 | 0.635 | 0.426 | 1 |
| 9 | rs2154380, rs1137101, rs4655537 | AAA | 0.402 | -0.634 | 1.22 | 0.269 | 1 |
|  |  | AAG | 0.0546 | 0.299 | 0.0543 | 0.816 | 1 |
|  |  | GGG | 0.541 | 0.318 | 0.315 | 0.575 | 1 |
| 10 | rs12405556, rs4606347, rs8179183 | CAC | 0.186 | 0.703 | 0.891 | 0.346 | 1 |
|  |  | AGG | 0.163 | -0.174 | 0.0525 | 0.819 | 1 |
|  |  | CGG | 0.65 | -0.314 | 0.282 | 0.596 | 1 |
| 11 | rs4567312, rs17415296, rs1805096, rs1892534 | GAAA | 0.0631 | 1.87 | 2.52 | 0.113 | 0.9738 |
|  |  | ACAA | 0.134 | 0.24 | 0.0875 | 0.767 | 1 |
|  |  | GCAA | 0.258 | -0.19 | 0.0812 | 0.776 | 1 |
|  |  | GCGG | 0.543 | -0.426 | 0.51 | 0.475 | 1 |
| *n*=848  Abbreviations: LEPR- leptin receptor gene, SNP- single nucleotide polymorphism, STAT- test statistic, BETA- regression coefficient | | | | | | | |

| ***LEPR* Haplotype Association Analysis of Fagerström Test for Nicotine Dependence Score in European American Group** | | | | | | | |
| --- | --- | --- | --- | --- | --- | --- | --- |
| **Block** | **SNPs** | **Haplotype** | **Frequency** | **BETA** | **STAT** | ***p*-value** | **Adjusted *p*-value** |
| 1 | rs3806318, rs12145690, rs9436297 | AAG | 0.135 | -0.131 | 0.263 | 0.608 | 1 |
|  |  | GCA | 0.263 | -0.172 | 0.738 | 0.391 | 0.9998 |
|  |  | ACA | 0.204 | -0.00178 | 6.55E-05 | 0.994 | 1 |
|  |  | AAA | 0.395 | 0.18 | 0.965 | 0.326 | 0.9994 |
| 2 | rs7534511, rs9436301, rs12065099, rs11208648, rs1887285, rs970467 | GGAAAA | 0.124 | 0.168 | 0.436 | 0.51 | 1 |
|  |  | GGAAGG | 0.0997 | 0.432 | 1.65 | 0.2 | 0.9896 |
|  |  | GAGGAG | 0.0501 | -0.354 | 0.602 | 0.438 | 1 |
|  |  | GGAAAG | 0.0355 | -0.382 | 0.732 | 0.393 | 1 |
|  |  | AAAAAG | 0.315 | -0.0924 | 0.254 | 0.615 | 1 |
|  |  | GAAAAG | 0.372 | -0.0114 | 0.00392 | 0.95 | 1 |
| 3 | rs9436748, rs6657868, rs6669354 | CGC | 0.128 | 0.252 | 1.02 | 0.314 | 0.9992 |
|  |  | CAA | 0.381 | -0.191 | 1.23 | 0.268 | 0.9976 |
|  |  | AGA | 0.399 | -0.0387 | 0.0483 | 0.826 | 1 |
|  |  | CGA | 0.0892 | 0.435 | 1.59 | 0.208 | 0.9918 |
| 4 | rs6588147, rs12409877, rs2025803, rs7513047, rs11808888, rs17127673, rs11579567, rs1327121, rs6694528, rs11208654, rs1327115, rs10889557, | AGGAGACAGACG | 0.444 | -0.03 | 0.0289 | 0.865 | 1 |
|  |  | AGAGAGCAAACG | 0.149 | 0.705 | 7.59 | 0.0062 | 0.1494 |
|  |  | GAAGGACGGGAA | 0.262 | -0.16 | 0.734 | 0.392 | 1 |
|  |  | GAAGGACGGGAG | 0.0624 | -0.0643 | 0.0327 | 0.857 | 1 |
|  |  | AAAGGAAAGAAG | 0.0609 | -0.635 | 2.67 | 0.103 | 0.8942 |
| 5 | rs11208659, rs1782755, rs1171278, rs6697315, rs10749753, rs1177681, rs12140910, rs10158279, rs3790427 | AAAAAAAAA | 0.155 | 0.303 | 1.52 | 0.218 | 0.9932 |
|  |  | ACGAGAAAA | 0.326 | -0.0532 | 0.0667 | 0.796 | 1 |
|  |  | ACGGAGACG | 0.249 | -0.161 | 0.699 | 0.404 | 1 |
|  |  | AAAAAAACA | 0.0241 | 1.11 | 3.1 | 0.079 | 0.8226 |
|  |  | ACGAGAACA | 0.125 | 0.00156 | 4.04E-05 | 0.995 | 1 |
|  |  | GCGGAAGCA | 0.0583 | -0.425 | 1.13 | 0.289 | 0.9988 |
|  |  | GCGGAAACA | 0.0237 | 0.134 | 0.0507 | 0.822 | 1 |
|  |  | AAAGAGACG | 0.0108 | 0.309 | 0.158 | 0.691 | 1 |
| 6 | rs3790426, rs11208675, rs10493380, rs12059300, rs6673591, rs1938489, rs12042877 | CAAGAAG | 0.318 | 0.2 | 1.13 | 0.289 | 0.9988 |
|  |  | CACGAAG | 0.188 | -0.0756 | 0.103 | 0.749 | 1 |
|  |  | CCAGGAA | 0.282 | -0.103 | 0.305 | 0.581 | 1 |
|  |  | AAAAGGG | 0.211 | -0.0453 | 0.0467 | 0.829 | 1 |
| 7 | rs2154380, rs1137101, rs4655537, rs12405556 | GGGA | 0.263 | -0.127 | 0.436 | 0.51 | 1 |
|  |  | AAAC | 0.341 | 0.171 | 0.854 | 0.356 | 0.9998 |
|  |  | GGGC | 0.195 | 0.00881 | 0.00169 | 0.967 | 1 |
|  |  | AAGC | 0.201 | -0.106 | 0.186 | 0.667 | 1 |
| 9 | rs4606347, rs8179183, rs6678033, rs17415296, rs1805096, rs1892534 | ACAAAA | 0.179 | -0.0437 | 0.0299 | 0.863 | 1 |
|  |  | GGACAA | 0.201 | 0.00745 | 0.00121 | 0.972 | 1 |
|  |  | GGGCGG | 0.604 | -0.0555 | 0.0873 | 0.768 | 1 |
| *n*=397  Abbreviations: LEPR- leptin receptor gene, SNP- single nucleotide polymorphism, STAT- test statistic, BETA- regression coefficient | | | | | | | |

| ***LEPR* Haplotype Association Analysis of Fagerström Test for Nicotine Dependence Score in African American Group** | | | | | | | |
| --- | --- | --- | --- | --- | --- | --- | --- |
| **Block** | **SNPs** | **Haplotype** | **Frequency** | **BETA** | **STAT** | ***p*-value** | **Adjusted *p*-value** |
| 1 | rs1214569, rs9436297 | AG | 0.139 | 0.297 | 1.59 | 0.208 | 0.9994 |
|  |  | CA | 0.42 | -0.0465 | 0.0863 | 0.769 | 1 |
|  |  | AA | 0.441 | -0.0879 | 0.307 | 0.58 | 1 |
| 2 | rs12077210, rs3790433, rs7534511, rs9436301, rs12065099, rs11208648, rs1887285, rs970467 | GGAAAAAG | 0.194 | -0.328 | 2.21 | 0.138 | 0.9888 |
|  |  | GAGGAAAA | 0.221 | 0.236 | 1.45 | 0.229 | 0.9998 |
|  |  | GAGGAAAG | 0.0486 | 0.496 | 1.62 | 0.204 | 0.9992 |
|  |  | GAGGAAGG | 0.122 | 0.379 | 2.05 | 0.153 | 0.9926 |
|  |  | GGGAAAAG | 0.0716 | 0.207 | 0.379 | 0.538 | 1 |
|  |  | AAGAGGAG | 0.285 | -0.26 | 1.88 | 0.171 | 0.9962 |
|  |  | GAGAGGAG | 0.0299 | -0.511 | 1.63 | 0.202 | 0.9992 |
|  |  | GAGAGGAA | 0.0108 | 1.04 | 1.89 | 0.17 | 0.9962 |
| 3 | rs9436746, rs9436748, rs6657868, rs6669354 | CCGC | 0.152 | 0.218 | 0.845 | 0.359 | 1 |
|  |  | ACAA | 0.472 | -0.301 | 3.66 | 0.0565 | 0.8498 |
|  |  | CAGA | 0.188 | 0.193 | 0.961 | 0.328 | 1 |
|  |  | CCGA | 0.0365 | -0.118 | 0.0769 | 0.782 | 1 |
|  |  | ACGA | 0.135 | 0.327 | 1.57 | 0.211 | 0.9994 |
| 4 | rs2025803, rs7513047, rs11808888, rs17127673, rs11579567, rs1327121, rs6694528, rs11208654, rs1327115 | AGGACGGGA | 0.201 | -0.388 | 3.32 | 0.0693 | 0.8978 |
|  |  | AGAGCAAAC | 0.329 | 0.163 | 0.871 | 0.351 | 1 |
|  |  | AGGAAAGAA | 0.157 | 0.0996 | 0.212 | 0.646 | 1 |
|  |  | GAGACAGAC | 0.0917 | 0.246 | 0.68 | 0.41 | 1 |
|  |  | AGAACAGAC | 0.0973 | 0.179 | 0.492 | 0.483 | 1 |
|  |  | AGAACAAAC | 0.0733 | -0.42 | 1.58 | 0.21 | 0.9994 |
|  |  | AGGACAGAA | 0.0309 | 0.0172 | 0.00125 | 0.972 | 1 |
| 5 | rs10889557, rs2767485 | GG | 0.292 | 0.271 | 2.35 | 0.126 | 0.9836 |
|  |  | AA | 0.412 | -0.261 | 2.62 | 0.107 | 0.97 |
|  |  | GA | 0.296 | 0.0377 | 0.0466 | 0.829 | 1 |
| 6 | rs1782755, rs1171278 | AA | 0.445 | 0.14 | 0.741 | 0.39 | 1 |
|  |  | CG | 0.553 | -0.14 | 0.741 | 0.39 | 1 |
| 7 | rs9660088, rs10158279, rs3790427, rs3790426, rs11208675, rs10493380, rs12059300, rs6673591 | ACAAAAAG | 0.109 | -0.287 | 1.2 | 0.274 | 0.9998 |
|  |  | GAACAAGA | 0.428 | 0.322 | 3.8 | 0.052 | 0.8276 |
|  |  | GCGCCAGG | 0.168 | -0.239 | 1.26 | 0.262 | 0.9998 |
|  |  | GCAAAAAG | 0.0876 | -0.136 | 0.241 | 0.624 | 1 |
|  |  | GAACACGA | 0.0552 | 0.225 | 0.371 | 0.543 | 1 |
|  |  | GCACCAGG | 0.0727 | -0.375 | 1.84 | 0.175 | 0.9972 |
|  |  | GCAAAAGG | 0.0172 | -0.278 | 0.139 | 0.71 | 1 |
|  |  | GCAACAGG | 0.0155 | 0.946 | 2.07 | 0.151 | 0.992 |
|  |  | GCACCCGG | 0.0121 | 0.123 | 0.0265 | 0.871 | 1 |
| 8 | rs1938489, rs12042877 | AA | 0.274 | -0.125 | 0.464 | 0.496 | 1 |
|  |  | GG | 0.226 | -0.324 | 2.79 | 0.0959 | 0.9596 |
|  |  | AG | 0.5 | 0.321 | 3.97 | 0.0471 | 0.7956 |
| 9 | rs2154380, rs1137101, rs4655537 | AAA | 0.402 | 0.185 | 1.18 | 0.278 | 0.9998 |
|  |  | AAG | 0.0546 | -0.189 | 0.276 | 0.6 | 1 |
|  |  | GGG | 0.541 | -0.173 | 1.09 | 0.296 | 1 |
| 10 | rs12405556, rs4606347, rs8179183 | CAC | 0.186 | -0.386 | 3.05 | 0.0814 | 0.9348 |
|  |  | AGG | 0.163 | -0.243 | 1.22 | 0.27 | 0.9998 |
|  |  | CGG | 0.65 | 0.378 | 4.97 | 0.0264 | 0.6057 |
| 11 | rs4567312, rs17415296, rs1805096, rs1892534 | GAAA | 0.0631 | 0.138 | 0.171 | 0.679 | 1 |
|  |  | ACAA | 0.134 | 0.0568 | 0.062 | 0.803 | 1 |
|  |  | GCAA | 0.258 | -0.136 | 0.493 | 0.483 | 1 |
|  |  | GCGG | 0.543 | 0.0336 | 0.0407 | 0.84 | 1 |
| *n*=404  Abbreviations: LEPR- leptin receptor gene, SNP- single nucleotide polymorphism, STAT- test statistic, BETA- regression coefficient | | | | | | | |

| ***LEPR* Haplotype Association Analysis of State-Trait Anxiety Inventory Trait Score in European American Group** | | | | | | | |
| --- | --- | --- | --- | --- | --- | --- | --- |
| **Block** | **SNPs** | **Haplotype** | **Frequency** | **BETA** | **STAT** | ***p*-value** | **Adjusted *p*-value** |
| 1 | rs3806318, rs12145690, rs9436297 | AAG | 0.135 | -0.0997 | 0.00671 | 0.935 | 1 |
|  |  | GCA | 0.263 | 0.922 | 1.12 | 0.291 | 0.9984 |
|  |  | ACA | 0.204 | 0.659 | 0.445 | 0.505 | 1 |
|  |  | AAA | 0.395 | -1.3 | 2.5 | 0.114 | 0.9226 |
| 2 | rs7534511, rs9436301, rs12065099, rs11208648, rs1887285, rs970467 | GGAAAA | 0.124 | -0.952 | 0.561 | 0.454 | 1 |
|  |  | GGAAGG | 0.0997 | 0.0837 | 0.00401 | 0.95 | 1 |
|  |  | GAGGAG | 0.0501 | -2.62 | 2.19 | 0.14 | 0.9586 |
|  |  | GGAAAG | 0.0355 | 4.92 | 5.04 | 0.0252 | 0.4533 |
|  |  | AAAAAG | 0.315 | 2.34 | 6.86 | 0.00903 | 0.2164 |
|  |  | GAAAAG | 0.372 | -1.78 | 4.55 | 0.0333 | 0.5423 |
| 3 | rs9436748, rs6657868, rs6669354 | CGC | 0.128 | 0.28 | 0.0512 | 0.821 | 1 |
|  |  | CAA | 0.381 | 1.97 | 5.47 | 0.0197 | 0.3921 |
|  |  | AGA | 0.399 | -2.08 | 6.39 | 0.0117 | 0.2675 |
|  |  | CGA | 0.0892 | -0.0812 | 0.00316 | 0.955 | 1 |
| 4 | rs6588147, rs12409877, rs2025803, rs7513047, rs11808888, rs17127673, rs11579567, rs1327121, rs6694528, rs11208654, rs1327115, rs10889557, | AGGAGACAGACG | 0.444 | -2.01 | 6.22 | 0.0129 | 0.2883 |
|  |  | AGAGAGCAAACG | 0.149 | 0.246 | 0.0449 | 0.832 | 1 |
|  |  | GAAGGACGGGAA | 0.262 | 2.89 | 9.13 | 0.00262 | 0.07119 |
|  |  | GAAGGACGGGAG | 0.0624 | 1.19 | 0.512 | 0.475 | 1 |
|  |  | AAAGGAAAGAAG | 0.0609 | -3.48 | 4.33 | 0.038 | 0.5883 |
| 5 | rs11208659, rs1782755, rs1171278, rs6697315, rs10749753, rs1177681, rs12140910, rs10158279, rs3790427 | AAAAAAAAA | 0.155 | 1.08 | 1.02 | 0.313 | 0.9992 |
|  |  | ACGAGAAAA | 0.326 | -2.5 | 8.07 | 0.00466 | 0.1254 |
|  |  | ACGGAGACG | 0.249 | 2.89 | 9.13 | 0.00263 | 0.07139 |
|  |  | AAAAAAACA | 0.0241 | -2.05 | 0.579 | 0.447 | 1 |
|  |  | ACGAGAACA | 0.125 | 0.485 | 0.164 | 0.686 | 1 |
|  |  | GCGGAAGCA | 0.0583 | -2.33 | 1.81 | 0.179 | 0.9834 |
|  |  | GCGGAAACA | 0.0237 | -1.58 | 0.429 | 0.513 | 1 |
|  |  | AAAGAGACG | 0.0108 | 8.71 | 2.24 | 0.135 | 0.9548 |
| 6 | rs3790426, rs11208675, rs10493380, rs12059300, rs6673591, rs1938489, rs12042877 | CAAGAAG | 0.318 | 0.25 | 0.0823 | 0.774 | 1 |
|  |  | CACGAAG | 0.188 | -3.11 | 9.34 | 0.00234 | 0.06379 |
|  |  | CCAGGAA | 0.282 | 2.73 | 8.35 | 0.00401 | 0.108 |
|  |  | AAAAGGG | 0.211 | -0.59 | 0.361 | 0.548 | 1 |
| 7 | rs2154380, rs1137101, rs4655537, rs12405556 | GGGA | 0.263 | 3.14 | 10.9 | 0.00102 | **0.03259** |
|  |  | AAAC | 0.341 | -1.11 | 1.68 | 0.196 | 0.9906 |
|  |  | GGGC | 0.195 | 0.0482 | 0.00232 | 0.962 | 1 |
|  |  | AAGC | 0.201 | -1.82 | 3.57 | 0.0593 | 0.7343 |
| 9 | rs4606347, rs8179183, rs6678033, rs17415296, rs1805096, rs1892534 | ACAAAA | 0.179 | -1.4 | 1.75 | 0.186 | 0.9866 |
|  |  | GGACAA | 0.201 | 3.56 | 11.8 | 0.00063 | **0.0218** |
|  |  | GGGCGG | 0.604 | -1.22 | 2.1 | 0.148 | 0.9672 |
| *n*=571  Abbreviations: LEPR- leptin receptor gene, SNP- single nucleotide polymorphism, STAT- test statistic, BETA- regression coefficient  Adjusted *p*-values in bold indicate statistically significant associations | | | | | | | |

| ***LEPR* Haplotype Association Analysis of State-Trait Anxiety Inventory Trait Score in African American Group** | | | | | | | |
| --- | --- | --- | --- | --- | --- | --- | --- |
| **Block** | **SNPs** | **Haplotype** | **Frequency** | **BETA** | **STAT** | ***p*-value** | **Adjusted *p*-value** |
| 1 | rs1214569, rs9436297 | AG | 0.139 | 1.66 | 3.02 | 0.0827 | 0.9352 |
|  |  | CA | 0.42 | -0.757 | 1.3 | 0.255 | 1 |
|  |  | AA | 0.441 | -0.0199 | 0.000847 | 0.977 | 1 |
| 2 | rs12077210, rs3790433, rs7534511, rs9436301, rs12065099, rs11208648, rs1887285, rs970467 | GGAAAAAG | 0.194 | -0.449 | 0.27 | 0.604 | 1 |
|  |  | GAGGAAAA | 0.221 | 1.14 | 2.05 | 0.152 | 0.9958 |
|  |  | GAGGAAAG | 0.0486 | 0.947 | 0.341 | 0.559 | 1 |
|  |  | GAGGAAGG | 0.122 | 0.919 | 0.731 | 0.393 | 1 |
|  |  | GGGAAAAG | 0.0716 | -1.61 | 1.33 | 0.249 | 1 |
|  |  | AAGAGGAG | 0.285 | -0.312 | 0.163 | 0.686 | 1 |
|  |  | GAGAGGAG | 0.0299 | -0.217 | 0.0143 | 0.905 | 1 |
|  |  | GAGAGGAA | 0.0108 | -0.726 | 0.0547 | 0.815 | 1 |
| 3 | rs9436746, rs9436748, rs6657868, rs6669354 | CCGC | 0.152 | 0.274 | 0.0861 | 0.769 | 1 |
|  |  | ACAA | 0.472 | -0.49 | 0.532 | 0.466 | 1 |
|  |  | CAGA | 0.188 | 0.759 | 0.766 | 0.382 | 1 |
|  |  | CCGA | 0.0365 | -1.2 | 0.442 | 0.506 | 1 |
|  |  | ACGA | 0.135 | 0.827 | 0.642 | 0.423 | 1 |
| 4 | rs2025803, rs7513047, rs11808888, rs17127673, rs11579567, rs1327121, rs6694528, rs11208654, rs1327115 | AGGACGGGA | 0.201 | -0.981 | 1.29 | 0.257 | 1 |
|  |  | AGAGCAAAC | 0.329 | -0.23 | 0.1 | 0.752 | 1 |
|  |  | AGGAAAGAA | 0.157 | 1.11 | 1.43 | 0.233 | 0.9998 |
|  |  | GAGACAGAC | 0.0917 | 1.42 | 1.36 | 0.243 | 1 |
|  |  | AGAACAGAC | 0.0973 | 0.698 | 0.373 | 0.541 | 1 |
|  |  | AGAACAAAC | 0.0733 | 0.214 | 0.0239 | 0.877 | 1 |
|  |  | AGGACAGAA | 0.0309 | -0.881 | 0.193 | 0.66 | 1 |
| 5 | rs10889557, rs2767485 | GG | 0.292 | 0.32 | 0.199 | 0.656 | 1 |
|  |  | AA | 0.412 | -1.61 | 5.53 | 0.0189 | 0.5073 |
|  |  | GA | 0.296 | 1.54 | 4.41 | 0.0362 | 0.7227 |
| 6 | rs1782755, rs1171278 | AA | 0.445 | -0.873 | 1.61 | 0.205 | 0.9992 |
|  |  | CG | 0.553 | 0.921 | 1.8 | 0.18 | 0.9978 |
| 7 | rs9660088, rs10158279, rs3790427, rs3790426, rs11208675, rs10493380, rs12059300, rs6673591 | ACAAAAAG | 0.109 | -3.32 | 9.09 | 0.00267 | 0.1054 |
|  |  | GAACAAGA | 0.428 | 1.28 | 3.67 | 0.056 | 0.8538 |
|  |  | GCGCCAGG | 0.168 | -1.47 | 3.01 | 0.0833 | 0.9364 |
|  |  | GCAAAAAG | 0.0876 | 0.953 | 0.639 | 0.425 | 1 |
|  |  | GAACACGA | 0.0552 | 2.28 | 2.61 | 0.107 | 0.9724 |
|  |  | GCACCAGG | 0.0727 | 0.057 | 0.0021 | 0.964 | 1 |
|  |  | GCAAAAGG | 0.0172 | 0.212 | 0.00763 | 0.93 | 1 |
|  |  | GCAACAGG | 0.0155 | -3.54 | 1.73 | 0.189 | 0.9982 |
|  |  | GCACCCGG | 0.0121 | 1.91 | 0.265 | 0.607 | 1 |
| 8 | rs1938489, rs12042877 | AA | 0.274 | -1.13 | 2.25 | 0.134 | 0.9902 |
|  |  | GG | 0.226 | -1.4 | 3.04 | 0.0816 | 0.9328 |
|  |  | AG | 0.5 | 1.73 | 7.27 | 0.00719 | 0.2386 |
| 9 | rs2154380, rs1137101, rs4655537 | AAA | 0.402 | 1.58 | 5.57 | 0.0185 | 0.4999 |
|  |  | AAG | 0.0546 | 1.21 | 0.649 | 0.421 | 1 |
|  |  | GGG | 0.541 | -1.71 | 6.78 | 0.00943 | 0.2979 |
| 10 | rs12405556, rs4606347, rs8179183 | CAC | 0.186 | -0.358 | 0.164 | 0.685 | 1 |
|  |  | AGG | 0.163 | -0.774 | 0.737 | 0.391 | 1 |
|  |  | CGG | 0.65 | 0.638 | 0.846 | 0.358 | 1 |
| 11 | rs4567312, rs17415296, rs1805096, rs1892534 | GAAA | 0.0631 | 2.65 | 3.7 | 0.0549 | 0.8488 |
|  |  | ACAA | 0.134 | 0.515 | 0.306 | 0.58 | 1 |
|  |  | GCAA | 0.258 | -1.68 | 4.41 | 0.0361 | 0.7223 |
|  |  | GCGG | 0.543 | 0.269 | 0.146 | 0.702 | 1 |
| *n*=642  Abbreviations: LEPR- leptin receptor gene, SNP- single nucleotide polymorphism, STAT- test statistic, BETA- regression coefficient | | | | | | | |

| ***LEPR* Haplotype Association Analysis of Montgomery-Asberg Depression Rating Scale Score in European American Group** | | | | | | | |
| --- | --- | --- | --- | --- | --- | --- | --- |
| **Block** | **SNPs** | **Haplotype** | **Frequency** | **BETA** | **STAT** | ***p*-value** | **Adjusted *p*-value** |
| 1 | rs3806318, rs12145690, rs9436297 | AAG | 0.135 | 0.415 | 0.339 | 0.56 | 1 |
|  |  | GCA | 0.263 | 0.495 | 0.8 | 0.372 | 1 |
|  |  | ACA | 0.204 | -0.564 | 0.833 | 0.362 | 1 |
|  |  | AAA | 0.395 | -0.274 | 0.294 | 0.588 | 1 |
| 2 | rs7534511, rs9436301, rs12065099, rs11208648, rs1887285, rs970467 | GGAAAA | 0.124 | 0.241 | 0.107 | 0.744 | 1 |
|  |  | GGAAGG | 0.0997 | -0.722 | 0.756 | 0.385 | 1 |
|  |  | GAGGAG | 0.0501 | -2.14 | 3.47 | 0.063 | 0.7542 |
|  |  | GGAAAG | 0.0355 | 2.53 | 3.55 | 0.06 | 0.7385 |
|  |  | AAAAAG | 0.315 | 0.109 | 0.041 | 0.84 | 1 |
|  |  | GAAAAG | 0.372 | 0.145 | 0.0796 | 0.778 | 1 |
| 3 | rs9436748, rs6657868, rs6669354 | CGC | 0.128 | 0.61 | 0.71 | 0.4 | 1 |
|  |  | CAA | 0.381 | 0.289 | 0.325 | 0.569 | 1 |
|  |  | AGA | 0.399 | -0.336 | 0.45 | 0.502 | 1 |
|  |  | CGA | 0.0892 | -0.971 | 1.17 | 0.279 | 0.9984 |
| 4 | rs6588147, rs12409877, rs2025803, rs7513047, rs11808888, rs17127673, rs11579567, rs1327121, rs6694528, rs11208654, rs1327115, rs10889557, | AGGAGACAGACG | 0.444 | -0.122 | 0.0596 | 0.807 | 1 |
|  |  | AGAGAGCAAACG | 0.149 | -0.307 | 0.189 | 0.664 | 1 |
|  |  | GAAGGACGGGAA | 0.262 | 0.653 | 1.36 | 0.244 | 0.996 |
|  |  | GAAGGACGGGAG | 0.0624 | 0.198 | 0.0371 | 0.847 | 1 |
|  |  | AAAGGAAAGAAG | 0.0609 | -1.75 | 2.68 | 0.102 | 0.8904 |
| 5 | rs11208659, rs1782755, rs1171278, rs6697315, rs10749753, rs1177681, rs12140910, rs10158279, rs3790427 | AAAAAAAAA | 0.155 | -0.376 | 0.312 | 0.577 | 1 |
|  |  | ACGAGAAAA | 0.326 | -0.43 | 0.608 | 0.436 | 1 |
|  |  | ACGGAGACG | 0.249 | 0.442 | 0.589 | 0.443 | 1 |
|  |  | AAAAAAACA | 0.0241 | 0.874 | 0.294 | 0.588 | 1 |
|  |  | ACGAGAACA | 0.125 | 0.844 | 1.35 | 0.245 | 0.996 |
|  |  | GCGGAAGCA | 0.0583 | -1.39 | 1.62 | 0.204 | 0.9892 |
|  |  | GCGGAAACA | 0.0237 | -1.23 | 0.618 | 0.432 | 1 |
|  |  | AAAGAGACG | 0.0108 | 0.421 | 0.0228 | 0.88 | 1 |
| 6 | rs3790426, rs11208675, rs10493380, rs12059300, rs6673591, rs1938489, rs12042877 | CAAGAAG | 0.318 | 0.183 | 0.114 | 0.736 | 1 |
|  |  | CACGAAG | 0.188 | -1.16 | 3.47 | 0.063 | 0.754 |
|  |  | CCAGGAA | 0.282 | 0.385 | 0.461 | 0.497 | 1 |
|  |  | AAAAGGG | 0.211 | 0.312 | 0.273 | 0.601 | 1 |
| 7 | rs2154380, rs1137101, rs4655537, rs12405556 | GGGA | 0.263 | 0.478 | 0.695 | 0.405 | 1 |
|  |  | AAAC | 0.341 | 0.146 | 0.0767 | 0.782 | 1 |
|  |  | GGGC | 0.195 | 0.283 | 0.21 | 0.647 | 1 |
|  |  | AAGC | 0.201 | -1.02 | 2.78 | 0.0961 | 0.875 |
| 9 | rs4606347, rs8179183, rs6678033, rs17415296, rs1805096, rs1892534 | ACAAAA | 0.179 | -0.995 | 2.34 | 0.126 | 0.9348 |
|  |  | GGACAA | 0.201 | 0.629 | 0.989 | 0.32 | 0.9996 |
|  |  | GGGCGG | 0.604 | 0.28 | 0.295 | 0.587 | 1 |
| *n*=830  Abbreviations: LEPR- leptin receptor gene, SNP- single nucleotide polymorphism, STAT- test statistic, BETA- regression coefficient | | | | | | | |

| ***LEPR* Haplotype Association Analysis of Montgomery-Asberg Depression Rating Scale Score in African American Group** | | | | | | | |
| --- | --- | --- | --- | --- | --- | --- | --- |
| **Block** | **SNPs** | **Haplotype** | **Frequency** | **BETA** | **STAT** | ***p*-value** | **Adjusted *p*-value** |
| 1 | rs1214569, rs9436297 | AG | 0.139 | 0.431 | 0.476 | 0.49 | 1 |
|  |  | CA | 0.42 | -0.244 | 0.341 | 0.559 | 1 |
|  |  | AA | 0.441 | 0.0703 | 0.0271 | 0.869 | 1 |
| 2 | rs12077210, rs3790433, rs7534511, rs9436301, rs12065099, rs11208648, rs1887285, rs970467 | GGAAAAAG | 0.194 | 0.101 | 0.0331 | 0.856 | 1 |
|  |  | GAGGAAAA | 0.221 | 0.236 | 0.212 | 0.645 | 1 |
|  |  | GAGGAAAG | 0.0486 | 0.246 | 0.0519 | 0.82 | 1 |
|  |  | GAGGAAGG | 0.122 | -0.191 | 0.0801 | 0.777 | 1 |
|  |  | GGGAAAAG | 0.0716 | -1.52 | 2.94 | 0.0867 | 0.945 |
|  |  | AAGAGGAG | 0.285 | 0.65 | 1.71 | 0.192 | 0.9978 |
|  |  | GAGAGGAG | 0.0299 | -0.448 | 0.15 | 0.699 | 1 |
|  |  | GAGAGGAA | 0.0108 | 0.173 | 0.00643 | 0.936 | 1 |
| 3 | rs9436746, rs9436748, rs6657868, rs6669354 | CCGC | 0.152 | -0.0458 | 0.00575 | 0.94 | 1 |
|  |  | ACAA | 0.472 | 0.73 | 2.9 | 0.0889 | 0.9482 |
|  |  | CAGA | 0.188 | -0.503 | 0.84 | 0.36 | 1 |
|  |  | CCGA | 0.0365 | -1.46 | 1.62 | 0.204 | 0.9988 |
|  |  | ACGA | 0.135 | 0.00623 | 9.14E-05 | 0.992 | 1 |
| 4 | rs2025803, rs7513047, rs11808888, rs17127673, rs11579567, rs1327121, rs6694528, rs11208654, rs1327115 | AGGACGGGA | 0.201 | -0.264 | 0.239 | 0.625 | 1 |
|  |  | AGAGCAAAC | 0.329 | -0.0562 | 0.0147 | 0.903 | 1 |
|  |  | AGGAAAGAA | 0.157 | 0.985 | 2.8 | 0.0945 | 0.9562 |
|  |  | GAGACAGAC | 0.0917 | -0.403 | 0.264 | 0.608 | 1 |
|  |  | AGAACAGAC | 0.0973 | 0.23 | 0.102 | 0.749 | 1 |
|  |  | AGAACAAAC | 0.0733 | -0.398 | 0.214 | 0.644 | 1 |
|  |  | AGGACAGAA | 0.0309 | 1.26 | 0.944 | 0.332 | 0.9998 |
| 5 | rs10889557, rs2767485 | GG | 0.292 | 0.212 | 0.209 | 0.647 | 1 |
|  |  | AA | 0.412 | -0.593 | 1.85 | 0.174 | 0.997 |
|  |  | GA | 0.296 | 0.456 | 0.917 | 0.339 | 0.9998 |
| 6 | rs1782755, rs1171278 | AA | 0.445 | -0.373 | 0.734 | 0.392 | 1 |
|  |  | CG | 0.553 | 0.418 | 0.926 | 0.336 | 0.9998 |
| 7 | rs9660088, rs10158279, rs3790427, rs3790426, rs11208675, rs10493380, rs12059300, rs6673591 | ACAAAAAG | 0.109 | -0.825 | 1.4 | 0.238 | 0.9998 |
|  |  | GAACAAGA | 0.428 | 0.572 | 1.73 | 0.188 | 0.9976 |
|  |  | GCGCCAGG | 0.168 | 0.395 | 0.529 | 0.467 | 1 |
|  |  | GCAAAAAG | 0.0876 | -0.743 | 0.958 | 0.328 | 0.9998 |
|  |  | GAACACGA | 0.0552 | 0.247 | 0.0714 | 0.789 | 1 |
|  |  | GCACCAGG | 0.0727 | -1.21 | 2.27 | 0.132 | 0.9872 |
|  |  | GCAAAAGG | 0.0172 | -1.41 | 0.651 | 0.42 | 1 |
|  |  | GCAACAGG | 0.0155 | -0.774 | 0.18 | 0.671 | 1 |
|  |  | GCACCCGG | 0.0121 | -0.646 | 0.0942 | 0.759 | 1 |
| 8 | rs1938489, rs12042877 | AA | 0.274 | -0.168 | 0.12 | 0.729 | 1 |
|  |  | GG | 0.226 | -0.855 | 2.74 | 0.098 | 0.9612 |
|  |  | AG | 0.5 | 0.693 | 2.73 | 0.0991 | 0.9628 |
| 9 | rs2154380, rs1137101, rs4655537 | AAA | 0.402 | 0.757 | 3.11 | 0.0784 | 0.9242 |
|  |  | AAG | 0.0546 | -0.811 | 0.697 | 0.404 | 1 |
|  |  | GGG | 0.541 | -0.546 | 1.66 | 0.199 | 0.9984 |
| 10 | rs12405556, rs4606347, rs8179183 | CAC | 0.186 | -0.459 | 0.669 | 0.414 | 1 |
|  |  | AGG | 0.163 | 0.578 | 1.03 | 0.31 | 0.9998 |
|  |  | CGG | 0.65 | -0.0569 | 0.0164 | 0.898 | 1 |
| 11 | rs4567312, rs17415296, rs1805096, rs1892534 | GAAA | 0.0631 | -0.0791 | 0.00788 | 0.929 | 1 |
|  |  | ACAA | 0.134 | 0.441 | 0.529 | 0.467 | 1 |
|  |  | GCAA | 0.258 | 0.0495 | 0.00955 | 0.922 | 1 |
|  |  | GCGG | 0.543 | -0.26 | 0.338 | 0.561 | 1 |
| *n*=815  Abbreviations: LEPR- leptin receptor gene, SNP- single nucleotide polymorphism, STAT- test statistic, BETA- regression coefficient | | | | | | | |

| ***LEPR* Haplotype Association Analysis of Body Mass Index in European American Group** | | | | | | | |
| --- | --- | --- | --- | --- | --- | --- | --- |
| **Block** | **SNPs** | **Haplotype** | **Frequency** | **BETA** | **STAT** | ***p*-value** | **Adjusted *p*-value** |
| 1 | rs3806318, rs12145690, rs9436297 | AAG | 0.135 | -0.184 | 0.443 | 0.506 | 1 |
|  |  | GCA | 0.263 | -0.0031 | 0.000227 | 0.988 | 1 |
|  |  | ACA | 0.204 | 0.174 | 0.568 | 0.451 | 1 |
|  |  | AAA | 0.395 | -0.0323 | 0.0284 | 0.866 | 1 |
| 2 | rs7534511, rs9436301, rs12065099, rs11208648, rs1887285, rs970467 | GGAAAA | 0.124 | -0.427 | 2.3 | 0.13 | 0.9416 |
|  |  | GGAAGG | 0.0997 | 0.414 | 1.76 | 0.184 | 0.9842 |
|  |  | GAGGAG | 0.0501 | -0.143 | 0.106 | 0.745 | 1 |
|  |  | GGAAAG | 0.0355 | 1.21 | 5.43 | 0.02 | 0.3805 |
|  |  | AAAAAG | 0.315 | -0.0695 | 0.12 | 0.729 | 1 |
|  |  | GAAAAG | 0.372 | -0.0569 | 0.0893 | 0.765 | 1 |
| 3 | rs9436748, rs6657868, rs6669354 | CGC | 0.128 | -0.527 | 3.63 | 0.0568 | 0.7239 |
|  |  | CAA | 0.381 | 0.083 | 0.188 | 0.665 | 1 |
|  |  | AGA | 0.399 | 0.0234 | 0.0156 | 0.901 | 1 |
|  |  | CGA | 0.0892 | 0.373 | 1.24 | 0.266 | 0.9986 |
| 4 | rs6588147, rs12409877, rs2025803, rs7513047, rs11808888, rs17127673, rs11579567, rs1327121, rs6694528, rs11208654, rs1327115, rs10889557, | AGGAGACAGACG | 0.444 | -0.0227 | 0.0148 | 0.903 | 1 |
|  |  | AGAGAGCAAACG | 0.149 | -0.185 | 0.477 | 0.49 | 1 |
|  |  | GAAGGACGGGAA | 0.262 | -0.0286 | 0.0184 | 0.892 | 1 |
|  |  | GAAGGACGGGAG | 0.0624 | 0.457 | 1.39 | 0.238 | 0.9956 |
|  |  | AAAGGAAAGAAG | 0.0609 | 0.234 | 0.331 | 0.565 | 1 |
| 5 | rs11208659, rs1782755, rs1171278, rs6697315, rs10749753, rs1177681, rs12140910, rs10158279, rs3790427 | AAAAAAAAA | 0.155 | 0.0521 | 0.0422 | 0.837 | 1 |
|  |  | ACGAGAAAA | 0.326 | -0.137 | 0.443 | 0.506 | 1 |
|  |  | ACGGAGACG | 0.249 | -0.0368 | 0.0292 | 0.864 | 1 |
|  |  | AAAAAAACA | 0.0241 | -0.434 | 0.472 | 0.492 | 1 |
|  |  | ACGAGAACA | 0.125 | 0.417 | 2.33 | 0.127 | 0.9388 |
|  |  | GCGGAAGCA | 0.0583 | 0.0567 | 0.0183 | 0.892 | 1 |
|  |  | GCGGAAACA | 0.0237 | -0.0317 | 0.00274 | 0.958 | 1 |
|  |  | AAAGAGACG | 0.0108 | -0.588 | 0.339 | 0.561 | 1 |
| 6 | rs3790426, rs11208675, rs10493380, rs12059300, rs6673591, rs1938489, rs12042877 | CAAGAAG | 0.318 | -0.207 | 1.05 | 0.306 | 0.9996 |
|  |  | CACGAAG | 0.188 | 0.106 | 0.202 | 0.653 | 1 |
|  |  | CCAGGAA | 0.282 | -0.03 | 0.0205 | 0.886 | 1 |
|  |  | AAAAGGG | 0.211 | 0.198 | 0.759 | 0.384 | 0.9998 |
| 7 | rs2154380, rs1137101, rs4655537, rs12405556 | GGGA | 0.263 | -0.0258 | 0.0147 | 0.903 | 1 |
|  |  | AAAC | 0.341 | -0.157 | 0.637 | 0.425 | 1 |
|  |  | GGGC | 0.195 | 0.0975 | 0.173 | 0.678 | 1 |
|  |  | AAGC | 0.201 | 0.151 | 0.433 | 0.511 | 1 |
| 9 | rs4606347, rs8179183, rs6678033, rs17415296, rs1805096, rs1892534 | ACAAAA | 0.179 | 0.108 | 0.196 | 0.658 | 1 |
|  |  | GGACAA | 0.201 | 0.0513 | 0.0481 | 0.826 | 1 |
|  |  | GGGCGG | 0.604 | -0.101 | 0.273 | 0.601 | 1 |
| *n*=1127  Abbreviations: LEPR- leptin receptor gene, SNP- single nucleotide polymorphism, STAT- test statistic, BETA- regression coefficient | | | | | | | |

| ***LEPR* Haplotype Association Analysis of Body Mass Index in African American Group** | | | | | | | |
| --- | --- | --- | --- | --- | --- | --- | --- |
| **Block** | **SNPs** | **Haplotype** | **Frequency** | **BETA** | **STAT** | ***p*-value** | **Adjusted *p*-value** |
| 1 | rs1214569, rs9436297 | AG | 0.139 | 0.471 | 1.72 | 0.19 | 0.9994 |
|  |  | CA | 0.42 | 0.0992 | 0.169 | 0.681 | 1 |
|  |  | AA | 0.441 | -0.318 | 1.69 | 0.193 | 0.9996 |
| 2 | rs12077210, rs3790433, rs7534511, rs9436301, rs12065099, rs11208648, rs1887285, rs970467 | GGAAAAAG | 0.194 | -0.262 | 0.674 | 0.412 | 1 |
|  |  | GAGGAAAA | 0.221 | -0.324 | 1.17 | 0.279 | 1 |
|  |  | GAGGAAAG | 0.0486 | -0.247 | 0.176 | 0.675 | 1 |
|  |  | GAGGAAGG | 0.122 | 0.0458 | 0.0147 | 0.904 | 1 |
|  |  | GGGAAAAG | 0.0716 | 0.713 | 2.03 | 0.155 | 0.9958 |
|  |  | AAGAGGAG | 0.285 | 0.26 | 0.846 | 0.358 | 1 |
|  |  | GAGAGGAG | 0.0299 | 0.0949 | 0.0183 | 0.893 | 1 |
|  |  | GAGAGGAA | 0.0108 | 0.694 | 0.308 | 0.579 | 1 |
| 3 | rs9436746, rs9436748, rs6657868, rs6669354 | CCGC | 0.152 | -0.547 | 2.38 | 0.123 | 0.9862 |
|  |  | ACAA | 0.472 | 0.182 | 0.55 | 0.458 | 1 |
|  |  | CAGA | 0.188 | 0.155 | 0.243 | 0.622 | 1 |
|  |  | CCGA | 0.0365 | -0.434 | 0.436 | 0.509 | 1 |
|  |  | ACGA | 0.135 | 0.215 | 0.348 | 0.556 | 1 |
| 4 | rs2025803, rs7513047, rs11808888, rs17127673, rs11579567, rs1327121, rs6694528, rs11208654, rs1327115 | AGGACGGGA | 0.201 | -0.455 | 2.08 | 0.149 | 0.995 |
|  |  | AGAGCAAAC | 0.329 | 0.324 | 1.5 | 0.22 | 1 |
|  |  | AGGAAAGAA | 0.157 | -0.566 | 2.75 | 0.0974 | 0.963 |
|  |  | GAGACAGAC | 0.0917 | 0.108 | 0.0599 | 0.807 | 1 |
|  |  | AGAACAGAC | 0.0973 | 0.459 | 1.24 | 0.265 | 1 |
|  |  | AGAACAAAC | 0.0733 | 0.241 | 0.249 | 0.618 | 1 |
|  |  | AGGACAGAA | 0.0309 | 0.336 | 0.204 | 0.651 | 1 |
| 5 | rs10889557, rs2767485 | GG | 0.292 | -0.0468 | 0.0309 | 0.86 | 1 |
|  |  | AA | 0.412 | 0.176 | 0.48 | 0.488 | 1 |
|  |  | GA | 0.296 | -0.152 | 0.316 | 0.574 | 1 |
| 6 | rs1782755, rs1171278 | AA | 0.445 | 0.285 | 1.31 | 0.253 | 1 |
|  |  | CG | 0.553 | -0.29 | 1.36 | 0.244 | 1 |
| 7 | rs9660088, rs10158279, rs3790427, rs3790426, rs11208675, rs10493380, rs12059300, rs6673591 | ACAAAAAG | 0.109 | 0.813 | 4 | 0.0458 | 0.7926 |
|  |  | GAACAAGA | 0.428 | -0.278 | 1.23 | 0.268 | 1 |
|  |  | GCGCCAGG | 0.168 | 0.0757 | 0.0584 | 0.809 | 1 |
|  |  | GCAAAAAG | 0.0876 | 0.482 | 1.21 | 0.272 | 1 |
|  |  | GAACACGA | 0.0552 | -0.542 | 1.01 | 0.314 | 1 |
|  |  | GCACCAGG | 0.0727 | -0.739 | 2.48 | 0.116 | 0.9814 |
|  |  | GCAAAAGG | 0.0172 | -0.977 | 1.02 | 0.313 | 1 |
|  |  | GCAACAGG | 0.0155 | -0.371 | 0.137 | 0.712 | 1 |
|  |  | GCACCCGG | 0.0121 | 1.62 | 1.92 | 0.166 | 0.9978 |
| 8 | rs1938489, rs12042877 | AA | 0.274 | -0.162 | 0.348 | 0.555 | 1 |
|  |  | GG | 0.226 | 0.605 | 4.2 | 0.0407 | 0.7534 |
|  |  | AG | 0.5 | -0.278 | 1.33 | 0.25 | 1 |
| 9 | rs2154380, rs1137101, rs4655537 | AAA | 0.402 | -0.043 | 0.0301 | 0.862 | 1 |
|  |  | AAG | 0.0546 | 0.377 | 0.451 | 0.502 | 1 |
|  |  | GGG | 0.541 | -0.0054 | 0.000487 | 0.982 | 1 |
| 10 | rs12405556, rs4606347, rs8179183 | CAC | 0.186 | 0.677 | 4.33 | 0.0378 | 0.7279 |
|  |  | AGG | 0.163 | -0.066 | 0.0398 | 0.842 | 1 |
|  |  | CGG | 0.65 | -0.403 | 2.43 | 0.119 | 0.9842 |
| 11 | rs4567312, rs17415296, rs1805096, rs1892534 | GAAA | 0.0631 | 0.194 | 0.143 | 0.706 | 1 |
|  |  | ACAA | 0.134 | 0.0924 | 0.0683 | 0.794 | 1 |
|  |  | GCAA | 0.258 | -0.293 | 1.02 | 0.313 | 1 |
|  |  | GCGG | 0.543 | 0.119 | 0.215 | 0.643 | 1 |
| *n*=930  Abbreviations: LEPR- leptin receptor gene, SNP- single nucleotide polymorphism, STAT- test statistic, BETA- regression coefficient | | | | | | | |
